# DeepSnap-Deep Learning Approach Predicts Progesterone Receptor Antagonist Activity With High Performance

**DOI:** 10.3389/fbioe.2019.00485

**Published:** 2020-01-22

**Authors:** Yasunari Matsuzaka, Yoshihiro Uesawa

**Affiliations:** Department of Medical Molecular Informatics, Meiji Pharmaceutical University, Tokyo, Japan

**Keywords:** chemical structure, progesterone receptor, DeepSnap, deep learning, QSAR, machine learning

## Abstract

The progesterone receptor (PR) is important therapeutic target for many malignancies and endocrine disorders due to its role in controlling ovulation and pregnancy via the reproductive cycle. Therefore, the modulation of PR activity using its agonists and antagonists is receiving increasing interest as novel treatment strategy. However, clinical trials using the PR modulators have not yet been found conclusive evidences. Recently, increasing evidence from several fields shows that the classification of chemical compounds, including agonists and antagonists, can be done with recent improvements in deep learning (DL) using deep neural network. Therefore, we recently proposed a novel DL-based quantitative structure-activity relationship (QSAR) strategy using transfer learning to build prediction models for agonists and antagonists. By employing this novel approach, referred as DeepSnap-DL method, which uses images captured from 3-dimension (3D) chemical structure with multiple angles as input data into the DL classification, we constructed prediction models of the PR antagonists in this study. Here, the DeepSnap-DL method showed a high performance prediction of the PR antagonists by optimization of some parameters and image adjustment from 3D-structures. Furthermore, comparison of the prediction models from this approach with conventional machine learnings (MLs) indicated the DeepSnap-DL method outperformed these MLs. Therefore, the models predicted by DeepSnap-DL would be powerful tool for not only QSAR field in predicting physiological and agonist/antagonist activities, toxicity, and molecular bindings; but also for identifying biological or pathological phenomena.

## Introduction

The progesterone receptor (PR: NCBI Gene ID:18667) is a member of the steroid receptor superfamily and plays essential roles in female reproductive events, such as the establishment and maintenance of pregnancy, menstrual cycle regulation, sexual behavior, and development of mammary glands. It is also responsible for developing the central nervous system by the regulation of cell proliferation and differentiation via a wide range of physiological process modulated by progesterone-mediated classical ligand-binding or non-classical novel non-genomic pathways (Garg et al., 2017; Leehy et al., 2018; Wu et al., 2018; Cenciarini and Proietti, 2019; González-Orozco and Camacho-Arroyo, 2019; Hawley and Mosura, 2019; Rudzinskas et al., 2019). In the clinic, steroidal PR agonists have been used in oral contraception and postmenopasusal hormone therapy (Fensome et al., 2005; Afhüppe et al., 2009; Lee et al., 2020). In addition, PR antagonists are gaining attention as a potential anti-cancer treatment due to their inhibitory effects on cell growth *in vitro*, affecting ovarian, breast, prostate, and bone cancer cells (Tieszen et al., 2011; Zheng et al., 2017; Ponikwicka-Tyszko et al., 2019; Ritch et al., 2019; Trabert et al., 2019). However, recent clinical trials on ovarian cancer with a selective progesterone receptor modulator, such as mifepristone, have largely been unsuccessful, despite high *in vitro* antagonist activity in nuclear PR (Rocereto et al., 2010; Ponikwicka-Tyszko et al., 2019). Recently, it has been shown that treatment of ovarian cancer with the progesterone agonist or antagonist may induce similar adverse effects, including tumor promotion, due to the absence of classical nuclear PRs in ovarian cancer (Ponikwicka-Tyszko et al., 2019). However, the classical nuclear PR antagonist activity is important in not only understanding other tumor propagation such as breast tumorigenesis through unique gene expression programme (Mohammed et al., 2015; Check, 2017), but also in regulation of the central and peripheral nervous systems (González-Orozco and Camacho-Arroyo, 2019).

*In silico* computational approaches such as machine learning (ML) methods are useful tools for discovery agonists and antagonists, particularly in modeling of ligand-binding protein activation with an increasing number of new chemical compounds synthesized (Banerjee et al., 2016; Niu et al., 2016; Asako and Uesawa, 2017; Wink et al., 2018; Bitencourt-Ferreira and de Azevedo, 2019; Da'adoosh et al., 2019; Kim G. B. et al., 2019). Among *in silico* approaches, both qualitative classification and quantitative prediction models by quantitative structure-activity relationship (QSAR) methods were reported using a large collection of environmental chemicals (Zang et al., 2013; Niu et al., 2016; Norinder and Boyer, 2016; Cotterill et al., 2019; Dreier et al., 2019; Heo et al., 2019). However, building high-performance prediction model requires specialized techniques, such as selecting appropriate features and algorithms (Beltran et al., 2018; Khan and Roy, 2018). In addition, the prediction results of the current model are often difficult to develop the drug discovery for clinical trials (Gayvert et al., 2016; Neves et al., 2018; Vamathevan et al., 2019). A deep learning (DL) approach with convolutional neural networks (CNNs), Rectified Linear Unit (ReLU), and max pooling is a promising, powerful tool for the classification modeling (Date and Kikuchi, 2018; Öztürk et al., 2018; Wang et al., 2018; Agajanian et al., 2019; Idakwo et al., 2019; Jo et al., 2019), where factors affecting its prediction performance include sufficient size, suitable representation, and accurate labeling of supervised input datasets (Bello et al., 2019; Chauhan et al., 2019; Liu P. et al., 2019). To resolve these issues, the DL-based QSAR modeling approach using molecular images produced by 3D chemical structure as input data was previously developed and referred to as the DeepSnap-DL approach (Uesawa, 2018). In addition, the Toxicology in the twenty-first Century (Tox21) 10k library, consisted of ~10,000 chemical structures, such as industrial chemicals, pesticides, natural food products, and drugs, contains corresponding endpoints of the quantitative high throughput screening to identify agonists and antagonists of signaling pathways by measuring reporter gene activities against these chemicals. This serves as a very useful resource when constructing the prediction model (Huang et al., 2014; Chen et al., 2015; Sipes et al., 2017; Cooper and Schürer, 2019). By utilizing datasets from this library, a lot of the prediction models for agonists and antagonist activities have been constructed and reported (Ribay et al., 2016; Asako and Uesawa, 2017; Balabin and Judson, 2018; Banerjee et al., 2018; Fernandez et al., 2018; Lynch et al., 2018, 2019; Bai et al., 2019; Idakwo et al., 2019; Matsuzaka and Uesawa, 2019b; Yuan et al., 2019; Zhang J. et al., 2019).

In this study, we evaluated the prediction performance of the PR antagonist activity by optimization the DL hyperparameters and adjusting 3D chemical structure preparation and input data size. Furthermore, we compared the performance between DeepSnap-DL and conventional MLs methods, such as random forest (RF), extreme gradient boosting (XGBoost, which we denote as XGB), and Light gradient boosting machine (LightGBM) with Bayesian optimization. We show the DeepSnap-DL method outperformed the three traditional MLs approaches. These findings suggest that the DeepSnap-DL approach may be applied to other protein agonist and antagonist activities with high-quality and high-throughput prediction.

## Results and Discussion

### Contributions of Splits of Dataset Angles in the DeepSnap-DL Approach for Prediction Performance

In order to analyze the influence of different splits for the training, validation, and test (Tra, Val, and Test) datasets and the angles when capturing Jmol-generated images in the DeepSnap approach, we randomly divided the input data of a total of 7,582 chemical compounds into five ratios, namely Tra:Val:Test = 1:1:1 to 5:5:1 (Table S1). A total of 25 prediction models, including five angles (120, 180, 240, 300, and 360°) and five dataset ratios of the Tra:Val:Test (1: 1: 1, 2: 2: 1, 3: 3: 1, 4: 4: 1, and 5: 5: 1) were build using 10-fold cross validation prepared randomly split. The results, for average loss in Val datasets: loss (Val), accuracy in Val datasets: Acc (Val), balanced accuracy: BAC, F, area under the curve: AUC, accuracy in test datasets: Acc (Test), and matthews correlation coefficient: MCC at 120 to 300° and five dataset ratios were ≤ 0.025, ≥ 99.3, ≥ 0.977, ≥ 0.906, ≥ 0.996, ≥ 0.979, and ≥ 0.898, respectively (Table 1, Table S2). However, at 360° angle, average loss (Val), Acc (Val), BAC, F, AUC, Acc (Test), and MCC for the five dataset ratios were ≤ 0.254, ≥ 92. 7, ≥ 0.781, ≥ 0.378, ≥ 0.855, ≥ 0.712, and ≥ 0.352, respectively (Table 1, Table S2). The five angles (120, 180, 240, 300, and 360°) produced 27, 8, 8, 8, and 1 picture(s), respectively, from the 3D structures using the DeepSnap approach. These results suggest that multiple pictures produced by the DeepSnap method outperformed single images derived from at 360° angle. In addition, to confirm that this very high prediction performance was not due to overfitting, a permutation test was conducted by PR antagonist-non-specific activity score labeling. The results, for average loss (Val), Acc (Val), BAC, F, AUC, Acc (Test), and MCC at 120 to 360° and five kinds of datasets ratio were 0.322 or less, 90.4 or less, 0.496 or less, 0.168 or less, 0.527 or less, 0.511 or less, −0.009 or less, respectively (Table 1, Table S2). These results suggested that the high-performances in the PR antagonist prediction models may not be overfitting with the datasets.

**Table 1:** Prediction performances with different dataset sizes and angles on the DeepSnap-Deep Learning.

	**Angles**	**120**°****		**180**°****		**240**°****		**300**°****		**360**°****	
	**tra:val:test**	**Means**	**SD**	**Means**	**SD**	**Means**	**SD**	**Means**	**SD**	**Means**	**SD**
AUC	1: 1: 1	0.996	0.004	0.997	0.002	0.997	0.001	0.996	0.002	0.855	0.012
AUC	2: 2: 1	0.997	0.003	0.996	0.002	0.997	0.002	0.996	0.003	0.874	0.016
AUC	3: 3: 1	0.999	0.001	0.999	0.001	0.999	0.001	0.998	0.001	0.905	0.020
AUC	4: 4: 1	0.999	0.001	0.998	0.002	0.999	0.001	0.999	0.001	0.911	0.025
AUC	5: 5: 1	0.997	0.002	0.998	0.002	0.998	0.001	0.998	0.001	0.909	0.017
AUC	5: 5: 1 PMT	0.519	0.028	0.527	0.019	0.527	0.025	0.527	0.014	0.526	0.019
Acc (Test)	1: 1: 1	0.984	0.007	0.982	0.007	0.982	0.006	0.981	0.008	0.712	0.027
Acc (Test)	2: 2: 1	0.985	0.011	0.981	0.009	0.983	0.006	0.979	0.008	0.747	0.025
Acc (Test)	3: 3: 1	0.985	0.007	0.990	0.005	0.986	0.004	0.983	0.011	0.812	0.042
Acc (Test)	4: 4: 1	0.987	0.008	0.986	0.006	0.990	0.005	0.988	0.007	0.836	0.045
Acc (Test)	5: 5: 1	0.989	0.006	0.987	0.008	0.983	0.009	0.981	0.012	0.814	0.055
Acc (Test)	5: 5: 1 PMT	0.408	0.208	0.511	0.183	0.412	0.179	0.457	0.212	0.426	0.193
MCC	1: 1: 1	0.924	0.028	0.911	0.030	0.911	0.026	0.907	0.036	0.352	0.015
MCC	2: 2: 1	0.924	0.049	0.905	0.040	0.914	0.028	0.898	0.035	0.391	0.026
MCC	3: 3: 1	0.927	0.032	0.946	0.025	0.927	0.018	0.916	0.046	0.462	0.053
MCC	4: 4: 1	0.932	0.038	0.930	0.029	0.947	0.022	0.938	0.034	0.489	0.067
MCC	5: 5: 1	0.942	0.028	0.935	0.036	0.917	0.040	0.909	0.050	0.489	0.063
MCC	5: 5: 1 PMT	−0.038	0.044	−0.012	0.067	−0.015	0.068	−0.030	0.101	−0.009	0.079

*Parameters (MPS:100, ZF:100, AT:23%, BR:14.5mÅ, BMD:0.4Å, BT:0.8Å, LR:0.0008, BS:108, GoogleNet)*.

*5:5:1 PMT showed permutation test using Tra:Val:Test = 5:5:1*.

*ZF, zoom factor; AT, atom size; BR, bond radius; BMD, minimum bond distance; BT, bond tolerance; LR, learning rate; BS, batch size*.

### Contributions of Combinations of Pictures From Different Angles in the DeepSnap-DL Approach for Prediction Performance

**Figure 1:**
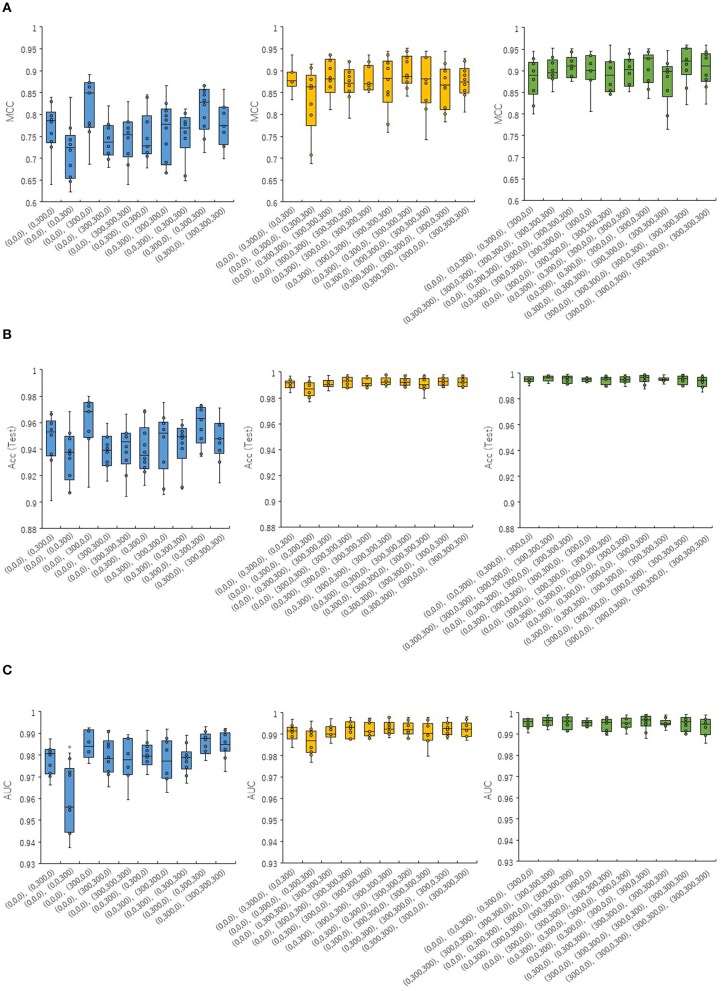
Prediction performances for combinations of different angles in DeepSnap. Two (blue boxes in right), three (yellow boxes in middle), and four (green boxes in left) of pictures were randomly selected from eight pictures produced at angle 300°, after which 10 kinds of picture combinations were prepared. The means of **(A)** MCC, **(B)** Acc(Test), and **(C)** AUC were calculated by 10-fold cross validation. *Pc < 0.01.

**Figure 2:**
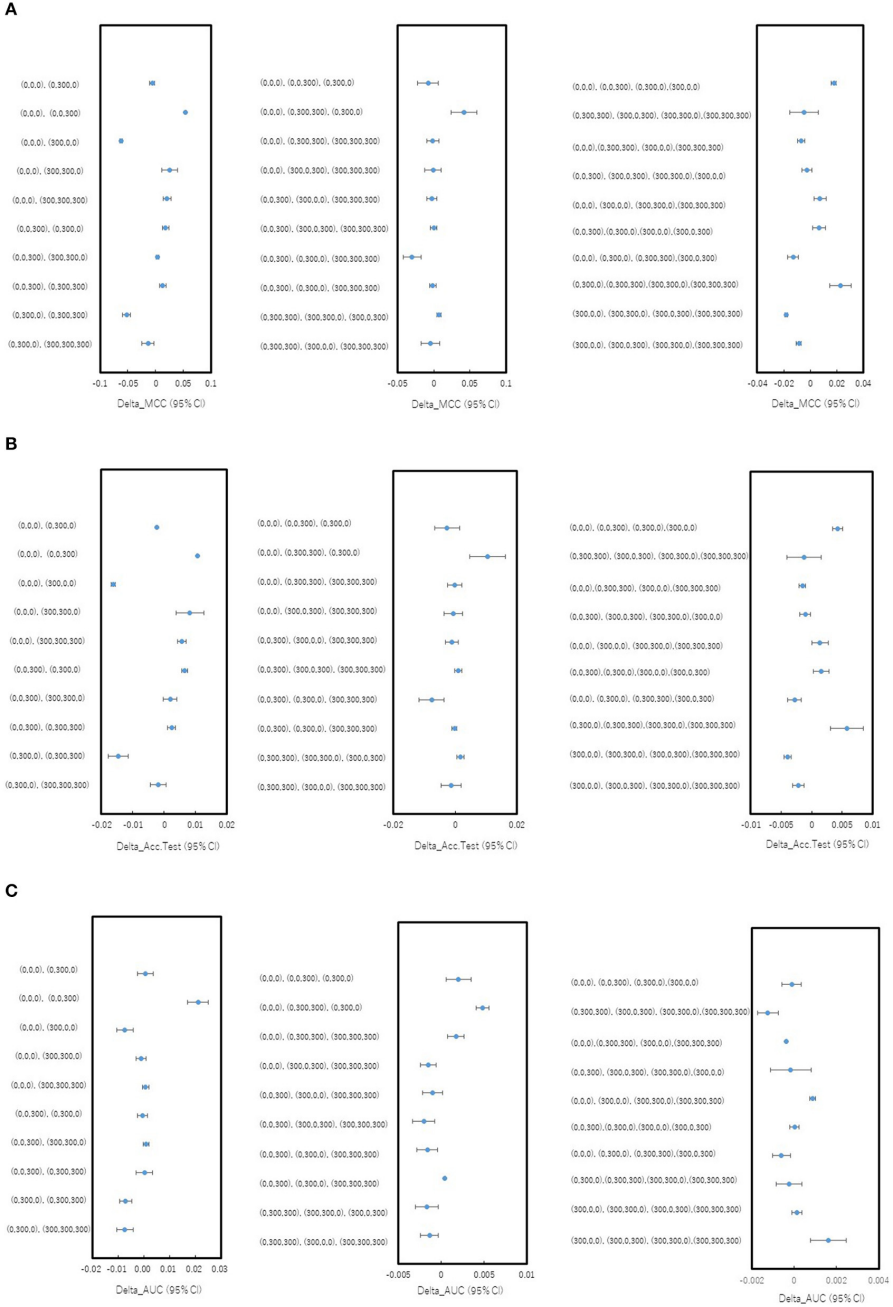
Differences in mean levels of performance for combinations of different angles in DeepSnap. Difference between mean levels of performance of one combination and rest nine combinations for pick-up pictures from eight pictures produced at angle 300° in Figure 1 were shown as blue dots with 95% confident interval (95% CI) as error bars. **(A)** Delta MCC (95% CI), **(B)** Delta Acc.Test (95% CI), and **(C)** Delta_AUC (95% CI) were calculated based on results in Figures 1A–C.

In order to investigate whether the combinations of pictures produced from different angles in the DeepSnap affect the prediction performance of the PR antagonist, two, three, or four pictures were randomly selected from eight pictures produced at 300° angle, which is small number of pictures produced in the DeepSnap and can be expected reduction of calculation cost. A total of 10 combinations of two, three, and four pick-up pictures each were used for building the prediction models using the DL method with a Tra:Val:Test ratio of 5:5:1. The performance of MCC, Acc (Test), AUC, BAC, F, Acc (Val), and Loss (Val) at two pictures was lower than those at three and four pictures (Figures 1A–C, Figures S1A–D). To compare these seven of indicators for performance between one and rest nine combinations among total 10 combinations, multiple comparison test was performed. The AUC and BAC at the two pictures combinations of [(0,0,0), (0,0,300)] indicated significantly lower results compared with those of the other nine combinations (Figure 1C, Figure S1A, Pc < 0.01). However, the MCC and Acc (Test) did not show significant differences for any combinations (Figures 1A,B). In addition, the Acc (Val) and Loss (Val) at two pictures combinations of [(0,0,0), (0,0,300)] were significantly higher and lower than those of other nine combinations (Figures S1C,D, Pc < 0.01). In addition, in order to show the differences of means of the performance indicator for one combination with means of the rest nine combinations, the nine delta values, which are difference of means of one and rest nine combinations and 95% confidence intervals (CIs) were examined (Figures 2A–C, Figures S2A–D). Two combinations of [(0,0,0), (0,0,300), and (0,0,0), (0,300,300), (0,300,0)] were showed high positive delta values (Figures 2A–C, Figures S2A,B). Combined, these results suggest that the combinations of pictures produced from different angles in DeepSnap may affect prediction performance and special combination of images with different angles may indicates high-performance.

### Contributions of Parameters for Prediction Performance in the DeepSnap-DL Approach

**Figure 3:**
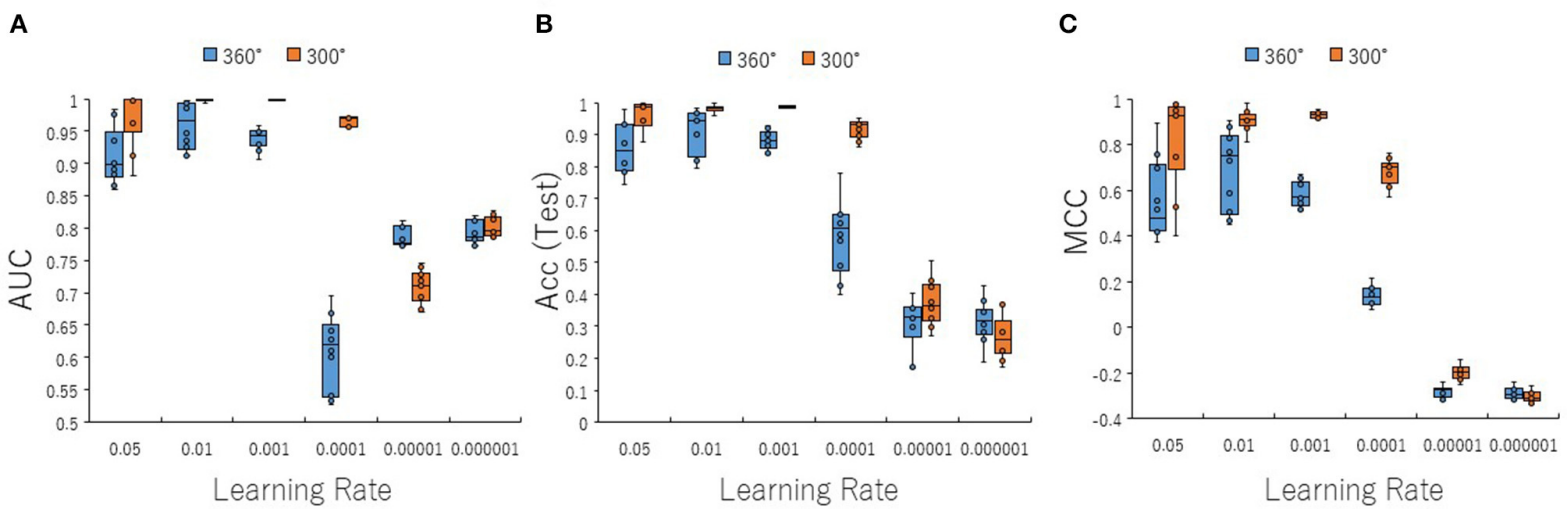
Performance contribution of prediction models with learning rates (LRs). The means of **(A)** AUC, **(B)** Acc(Test), and **(C)** MCC were calculated for six LRs from 0.05 to 0.000001 by 10-fold cross validation in the DeepSnap-DL-build prediction models using image produced by DeepSnap with two angles, 300° and 360°, with a Tra:Val:Test ratio of 5:5:1.

**Figure 4:**
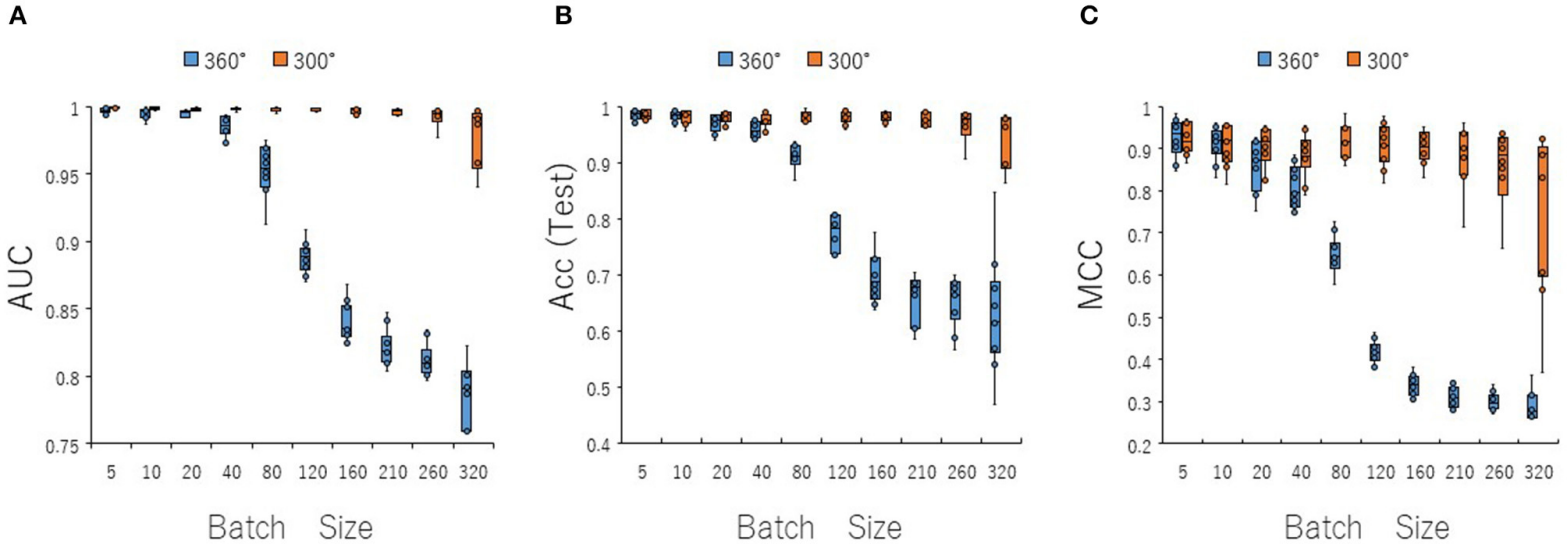
Performance contribution of prediction models with batch sizes (BSs). The means of **(A)** AUC, **(B)** Acc(Test), and **(C)** MCC were calculated for ten BSs from 5 to 320 by 10-fold cross validation in the DeepSnap-DL-build prediction models using images produced by DeepSnap for two angles, 300°and 360°, with a Tra:Val:Test ratio of 5:5:1.

**Figure 5:**
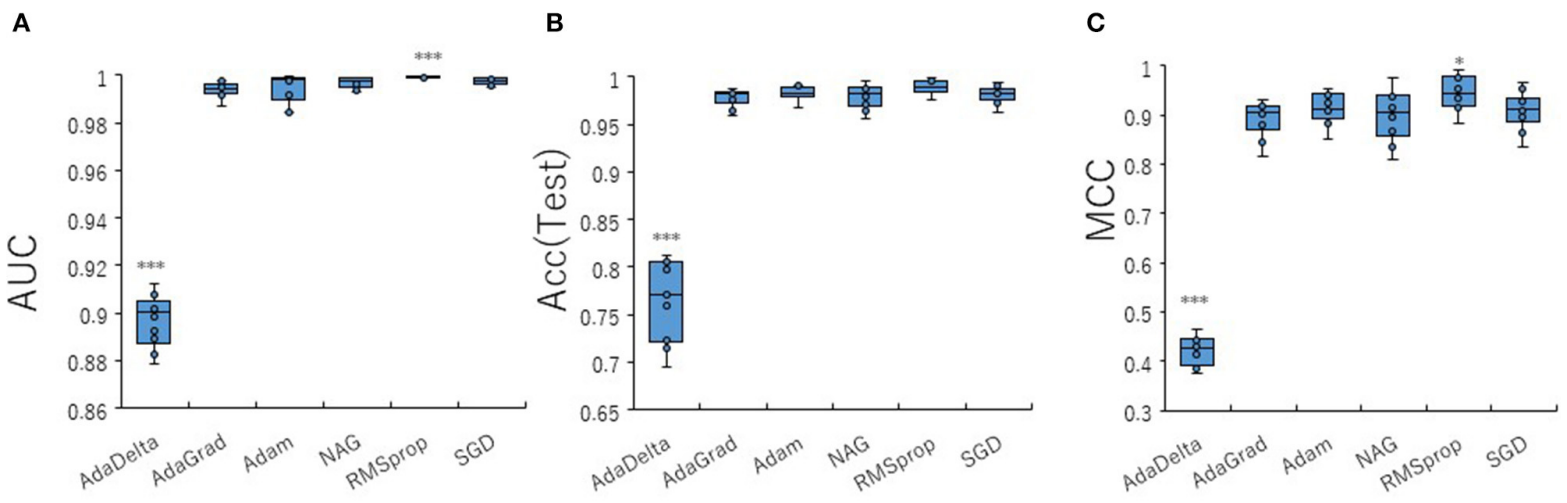
Performance contribution of prediction models with solver types (STs). The means of **(A)** AUC, **(B)** Acc(Test), and **(C)** MCC were calculated for six STs (AdaDelta, AdaGrad, Adam, NAG, RMSprop, and SGD) by 10-fold cross validation in the DeepSnap-DL-build prediction models using images produced by DeepSnap with angle 300° with a Tra:Val:Test ratio of 5:5:1. *Pc < 0.05, ***Pc < 0.001.

**Figure 6:**
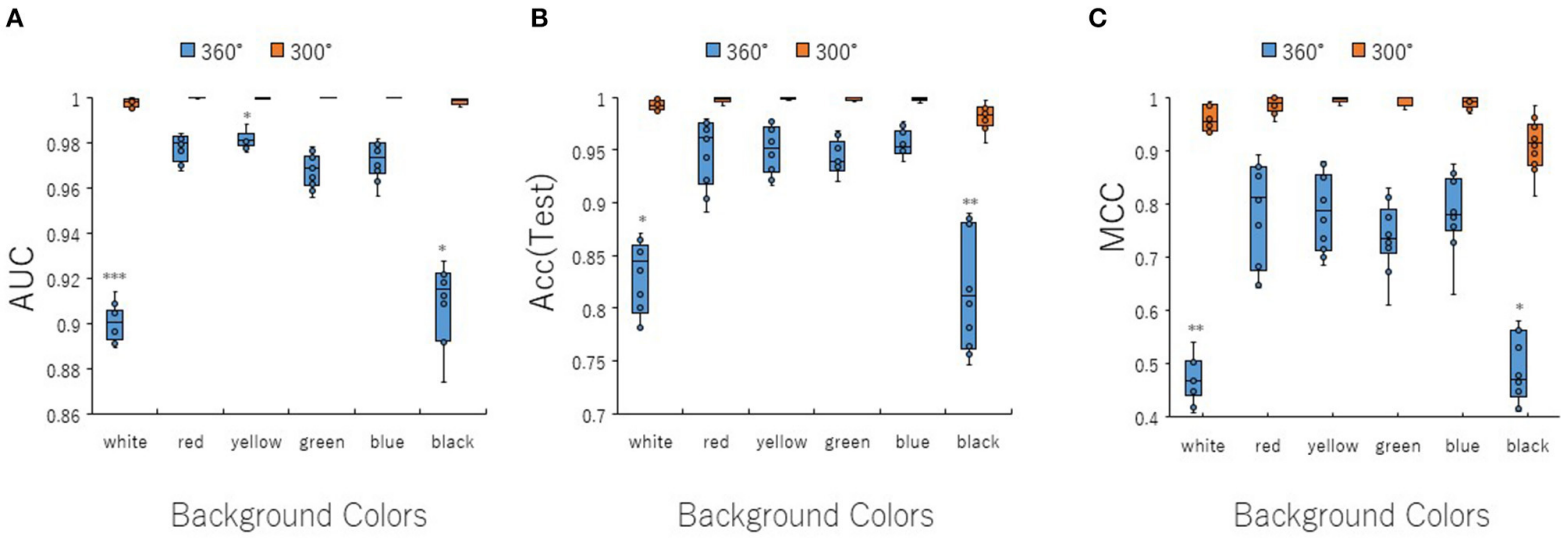
Performance contribution of prediction models with background image colors. The means of **(A)** AUC, **(B)** Acc(Test), and **(C)** MCC were calculated for six background colors (white, red, yellow, green, blue, and black) of image pictures produced by DeepSnap for angles 300 and 360° by 10-fold cross validation in the DeepSnap-DL-build prediction models with a Tra:Val:Test ratio of 5:5:1. *Pc < 0.05, **Pc < 0.01, ***Pc < 0.001.

To optimize prediction performance of the PR antagonist, we analyzed three kinds of hyperparameters, including learning rates (LRs), batch sizes (BSs), and solver types (STs) in the DL process using a Tra:Val:Test ratio of 5:5:1. By employing two angles, at 300° and 360°, which correspond to eight and one image(s) per one chemical structure of images captured in DeepSnap, we studies a total of six LRs from 0.05 to 0.000001 of hyperparameter range in the DL that were fine-tuned by 10-fold cross validation (Figures 3A–C, Figures S3A–D). In this study, the highest prediction performance at 360° angle was observed on at LR:0.01, which indicated that the mean MCC, Acc (Test), AUC, Loss(Val), Acc(Val), F, and BAC were 0.689 ± 0.173, 0.910 ± 0.079, 0.959 ± 0.043, 0.016 ± 0.104, 99.72 ± 3.64, 0.705 ± 0.168, and 0.911 ± 0.063, respectively (Figures 3A–C, Figures S3A–D). Coversely, at 300° angle, the highest performance showed that the mean MCC, Acc (Test), F, and BAC at LR: 0.001 were 0.934 ± 0.173, 0.987 ± 0.072, 0.940 ± 0.167, and 0.981 ± 0.057, respectively, and the mean AUC, Loss(Val), and Acc(Val) at LR: 0.01 were 0.998 ± 0.038, 0.011 ±0.094, and 99.71 ± 3.54 respectively (Figures 3A–C, Figures S3A–D). These findings suggest that the optimal range of the LR may be from 0.01 to 0.001. Using the same method, a total of 10 BSs from 5 to 320 were used for optimization of hyperparameters in the DeepSnap-DL method at two kinds of angles, at 300° and 360°. The prediction performance at the two angles decreased with increasing BSs, and the 300° angle showed a higher performance compared with a 360° angle (Figures 4A–C, Figures S4A–D). The higher prediction performances at 300° and 360° angles indicated that the mean MCC, Acc (Test), AUC, Loss(Val), Acc(Val), F, and BAC at BS:5 were 0.924 ± 0.039 and 0.925 ± 0.046 for the mean MCCs at 300° and 360° angles, 0.985 ± 0.009 and 0.985 ± 0.010 for the mean Acc(Test)s at 300° and 360° angles, 0.999 ± 0.001 and 0.997 ± 0.002 for the mean AUCs at 300° and 360° angles, 0.012 ± 0.003 and 0.014 ± 0.004 for the mean Loss(Val)s at 300° and 360° angles, 99.70 ± 0.055 and 99.63 ± 0.203 for the mean Acc(Val)s at 300° and 360° angles, 0.930 ± 0.037 and 0.931 ± 0.044 for the mean Fs at 300° and 360° angles, and 0.989 ± 0.006 and 0.979 ± 0.005 for the mean BACs at 300° and 360° angles, respectively (Figures 4A–C, Figures S4A–D). There are tensions among the BS, the LR, and the learning speed and stability (Brownlee, 2018; Hoffer et al., 2018; Smith et al., 2018; Shallue et al., 2019). These results show that calculation speed may be reduced by further optimization of the interractiona among the BS, the LR, and other parameters. Further, a total of six STs including Adaptive Delta (AdaDelta), Adaptive gradient (AdaGrad), Adaptive Moment Estimation (Adam), Nesterv's accelerated gradient (NAG), Root Mean Square propagation (RMSprop), and Stochastic gradient descent (SGD) of hyperparameters in the DL were analyzed for prediction performance at 300° angle in the DeepSnap-DL approach. The prediction performances used the AdaDelta and RMSprop indicated a significant decrease and increase compared with other five STs, respectively (Figures 5A–C, Figures S5A–D). The AdaGrad calculates the mean of the gradient (Duchi et al., 2011), but the RMSprop calculates the exponential moving average of the square of the gradient (Tieleman and Hinton, 2012) so that the LR when building our prediction model may be adjusted according to the degree of the more recent parameter update. In this study, a pre-trained GoogLeNet was used as DL-based argorithms. Consistent with the our results, it was repored the high classification performance using GoogLeNet model pre-trained on Image Net as a feature extractor (Zhu et al., 2019). The deep neural networks (DNNs) are trained using the optimized SGD algorithm, which calculates a expected error gradient for the current model state by the training datasets, corrects the weights of a node in the network each time by backpropagation, where the amount of weight updated during the training is a configurable hyperparameter and called the LR (Mostafa et al., 2018; Zhao et al., 2019). The performance of the SGD depended on how LRs, which controls the rate or speed at the end of each batch of trainings are turned over time (Zhao et al., 2019). In general, when the LR is too large, weight updates will be diverse by increase of inadvertent gradient descent, resulted in osillated performance by a positive feedback loop (Bengio, 2012; Brownlee, 2018). On the other hand, when the LR is to small, wight updates with a high training error will be stuck with a slow learning speed. Therefore, it is important to find optomal LR for the modeling with high-performance (Bengio, 2012; Brownlee, 2018). However, it is impossible to estimate the optimal LR on a given dataset a priori. In addtion, when using probabilitistic gradient descent internally such as DL, the input dataset split into several subsets, whose numbers of training detaset used in the calculation of the error gradient before the weight update is a hyperparameter for the learning algorithm called the BS, due to the lessening of the influence of outliers during training (Balles et al., 2017; Brownlee, 2018). Consisten with previous report that a covariance of the update width of weight increases with the reduction of the BSs, and performance is improved by making it easier to converge to a flat local solution (Keskar et al., 2017; Brownlee, 2018), the prediction performance in this study was also increased with reduction of BSs of the SGD. It has been shown that small BS stimulates a regularizing effects and lower generalization error by adding a noisy (Li Y. et al., 2019; Wen et al., 2019). In this study, the error backpropagation for the training and the gradient descent method for the weight update on this DL were used, where the optimal solution that is the smallest error is leaded by adjusting the range of amount of repetitive weight updata based on the relationship that when the current value is close to the supervised data, the error becames small. In order not to fall into a non-optimized local solution, the LRs at the beginning of the present study is increased, and then decreased with the weight update at the end of the fine-tine. Futhermore, the performance in this study was improved by using small BSs, while calculation cost and memory usage were increased due to update of the weights in each units of the mini-batch. However, it was reported that the use of multi-core learning by rejecting unnecessary weights selection indicates better efficiency and shorter trining time (Połap et al., 2018). To assesse the contribution of the background colors of images produced by the DeepSnap method with the prediction performance, we then used a total of six color types, including white, red, yellow, green, blue, and black for both 300° and 360° in the DeepSnap-DL approach. The prdiction models built by the two background colors, including white and black, showed significantly low performance compared with the other four background colors at 360° angle (Figures 6A–C, Figures S6A–D). Conversely, six background colors at 300° angle indicated high performance, but white and black colors showed slightly lower performances compared with the other four background colors (Figures 6A–C, Figures S6A–D). These results suggest that the DeepSnap-DL method could improve the prediction performance via parameter optimization.

### Contributions of Conformational Sampling of Chemical Compounds for Prediction Performance of the PR Antagonist in the DeepSnap-DL Approach

**Figure 7:**
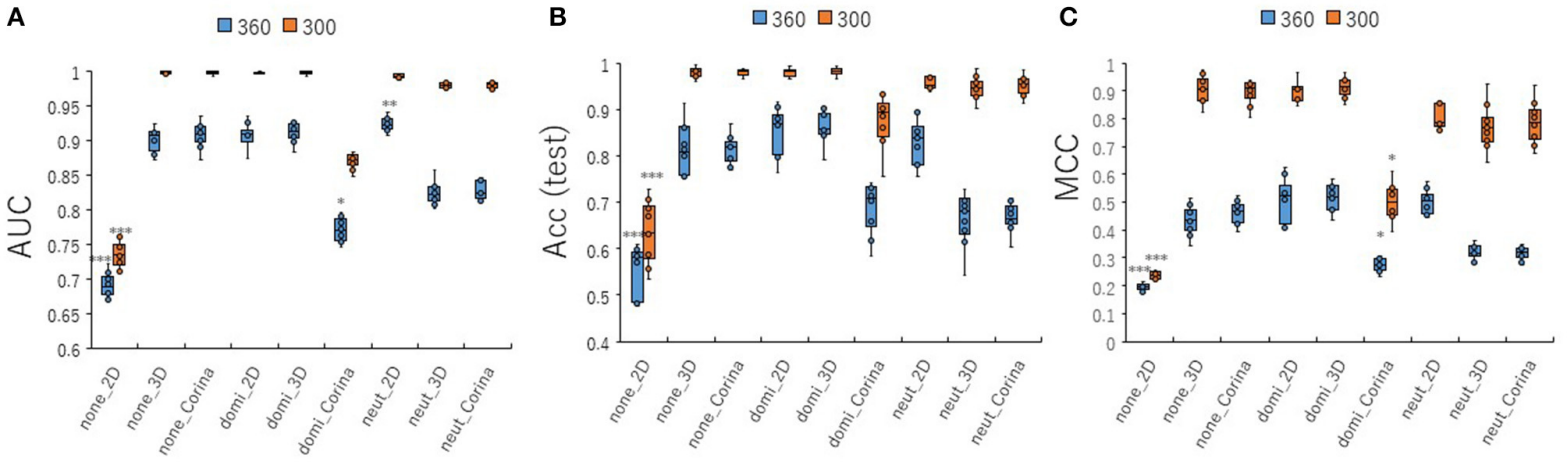
Performance contribution of prediction models with different wash conditions for preparation of chemical structures using molecular operating environment (MOE) software. For the preparation of 3D chemical structures by MOE software, combinations of three kinds of protonation (none, dominate, neutralize) and three kinds of coordinates (2D, 3D, CORINA) were used. The means of **(A)** AUC, **(B)** Acc(Test), and **(C)** MCC were calculated for nine combinations of wash conditions (none_2D, none_3D, none_Corina, domi_2D, domi_3D, domi_Corina, neut_2D, neut_3D, and neut_Corina) for images produced by DeepSnap for two angles, 300°and 360°, by 10-fold cross validation with a Tra:Val:Test ratio of 5:5:1. *Pc < 0.05, **Pc < 0.01, ***Pc < 0.001.

To investigate the contribution of conformational sampling of the 3D- chemical structures to the prediction performance of the PR antagonist, the 3D structures were produced by the combinations of its cleaning rules by adjusting the three of protonation states (none, dominant, neutralize) and three coordinating washed species (depict 2D, rebuild 3D, CORINA) in wash treatment of the MOE software at two angles (300 and 360°). A total of nine cleaning rules by combining three protonation states and the three coordinating washed species was used to build the prediction models with a Tra:Val:Test ratio of 5:5:1 (Figures 7A–C, Figures S7A–D). All nine prediction performances at 300°evaluated by AUC, Acc(Test), MCC, BAC, and F were higher than those at 360° (Figures 7A–C, Figures S7A,B). Of the nine cleaning rules, the none_2D, which indicates protonation: none and coordinating washed specie: depict 2D at two angles, 300 and 360°, showed lowest prediction performance compared with those of any other eight combinations (Figures 7A–C, Figures S7A,B). In addition, five combinations, including none_3D, none_Corina, domi_2D, domi_3D, and neut_2D, showed the highest prediction performances compared with the other four combinations (Figures 7A–C, Figures S7A,B). These findings suggested the conformational sampling of the 3D- chemical structures may be a critical step for improving prediction performance of the PR antagonist.

### Comparison of the Prediction Performance of the PR Antagonists by the DeepSnap-DL Approach With the Conventional MLs

To compare the prediction performance of the DeepSnap approach with conventional MLs, three ML approaches, random forest (RF), XGBoost (XGB), and LightGBM (LGB) were used to build the prediction models of the PR antagonists by applying 7,581 of the 3D- chemical structures, which excluded one chemical (Sodium hexafluorosilicate; PubChem SID: 144212628) from 7,582 chemicals used in DeepSnap-DL method due to not enable to be adapted for application of descriptor extraction, to extract molecular descriptors using by a non-copy left open-source software application, MORDRED. A total of 687 descriptors were extracted from nine SDF files produced by nine cleaning rules, including three protonation states and three coordinating washed species. Principal component analysis (PCA) calculated eigenvalues, contribution rates (CRs), and Cumulative CRs of each Principal component (PC) for the nine cleaning rules (none_2D, none_3D, none_Corina, domi_2D, domi_3D, domi_ Corina, neut_2D, neut_3D and neut_ Corina) (Figures S8A–C). The eigenvalues, contribution rates (CRs), and Cumulative CRs of the nine cleaning rules indicated no differences (Figures S8A–C). The means CRs of PC1 and PC2 of the nine cleaning rules were ~26.7 ± 0.031 and 5.06 ± 0.003%, respectively (Figures S8A–C). Cumulative CRs from PC1 to PC10 were ~50.5 ± 0.059% (Figure S8C). In addition, clustering analysis of variables in the PCA were performed using descriptors extracted from the nine cleaning rules, and calculated number of variables belonging to the cluster (variables No.), cluster representative variable with the largest square of correlation coefficient with cluster component (variables), the percentage of fluctuation explained by their first PC of the fluctuations of variables belonging to the cluster (Fluctuation in Cluster), and percentage of total variation explained by each cluster component (Fluctuation in Total). All variables of the molecular descriptors were summarized in cluster by grouping similar variables, in which the top 15 for the overall percentage of explained variation were listed (Table S3). The means and total number of variables in each 15 of clusters were 16.7 ± 10.1 and 235 in none_2D, 15.4 ± 8.6 and 231 in none_3D, 15.5 ± 9.6 and 232 in none_Corina, 15.5 ± 9.6 and 232 in domi_2D, 15.2 ± 8.3 and 228 in domi_3D, 15.4 ± 8.6 and 231 in domi_ Corina, 15.5 ± 9.6 and 232 in neut_2D, 15.5 ± 9.6 and 232 in neut_3D, and 15.5 ± 9.6 and 232 in neut_ Corina, respectively (Table S3). The means and top of Fluctuation rates in each 15 of clusters were 0.828 ± 0.088 and 0.920 in none_2D, 0.833 ± 0.087 and 0.905 in none_3D, 0.839 ± 0.078 and 0.901 in none_Corina, 0.839 ± 0.078 and 0.901 in domi_2D, 0.838 ± 0.081 and 0.916 in domi_3D, 0.834 ± 0.081 and 0.908 in domi_ Corina, 0.839 ± 0.078 and 0.901 in neut_2D, 0.847 ± 0.072 and 0.901 in neut_3D, and 0.839 ± 0.078 and 0.901 in neut_ Corina (Table S3). The means and total of Fluctuation rates in each 15 of clusters were 0.022 ± 0.016 and 0.333 in none_2D, 0.022 ± 0.015 and 0.332 in none_3D, 0.022 ± 0.015 and 0.332 in none_Corina, 0.022 ± 0.015 and 0.332 in domi_2D, 0.022 ± 0.013 and 0.328 in domi_3D, 0.022 ± 0.014 and 0.329 in domi_ Corina, 0.022 ± 0.015 and 0.332 in neut_2D, 0.022 ± 0.015 and 0.332 in neut_3D, and 0.022 ± 0.015 and 0.332 in neut_ Corina (Table S3). Three kinds of MLs, including RF, XGB, and LGB, were applied to predict compound activity of the PR antagonists and build a total of nine prediction models of RF, XGB, and LGB, respectively, for three Tra:Test ratios (0.5:0.5, 0.8:0.2, and 0.9: 0.1) (Table 2). The highest mean AUC values for RF, XGB, and LGB in five independent tests that randomly split by sklearn.model_selection were observed for the domi_3D cleaning rule in the three test dataset sizes (Table 2). In addition, consistent with recent reports (Zhang J. et al., 2019), out of three MLs, the LGB showed the highest means AUC values in five independent tests compared with RF and XGB in the three test dataset sizes: 0.9267 ± 0.0047 (test dataset size = 0.5), 0.9309 ± 0.0093 (test dataset size = 0.2), and 0.9407 ± 0.0134 (test dataset size = 0.1) (Table 2). Recent research shows the performance and speed of the LGB algorithm are mainly determined by parameters and sample size (Zhang J. et al., 2019). Therefore, in order to improve the prediction performance of the PR antagonist, the LGB were applied with optimized parameters using a Bayesian hyperparameter optimization algorithm from the HyperOpt package. A total of 27 prediction models for the nine and three cleaning rules and test dataset sizes, respectively, were built using the LGB. Similarly, the domi_3D cleaning rule showed the highest performance compared with the other eight cleaning rules via HyperOpt optimization, which allows faster and more robust parameter optimization compared to wither grid or random search (Bergstra et al., 2011; Zhang J. et al., 2019) in all three of the test dataset sizes (Table 2). Furthermore, improved prediction performance of the domi_3D by HyperOpt was observed in two of the test dataset sizes, 0.9346 ± 0.0069 (test dataset size = 0.2), and 0.9411 ± 0.0126 (test dataset size = 0.1) (Table 2). However, the highest means AUCs optimized by HyperOpt were lower than mean AUC of the domi_3D prepared from 300° using the DeepSnap-DL approach (AUC = 0.9971 ± 0.0021). In, addition, to directly compare the performance between DeepSnap-DL and conventional MLs using same input data, the molecular descriptors were extracted from Tra and Test datasets (tra:test = 1:1 to 5:1) used in DeepSnap-DL by Moerdred. The means of number of the molecular descriptors used in MLs were 690 ± 6.85 in tra:test = 1:1, 689 ± 7.24 in tra:test = 2:1, 694 ± 14.58 in tra:test = 3:1, 690 ± 6.82 in tra:test = 4:1, 692 ± 9.34 in tra:test = 5:1, 709 ± 0.00 in tra:test = 5:1 PMT, respectively (Table 3). Four kinds of MLs, including RF, XGB, LGB, and CatBoost (CB) were applied to construct prediction models of compound activity of the PR antagonists using these molecular descriptors by 10-fold cross validation. The highest mean AUC values for RF, XGB, LGB, and CB in 10-fold cross validation were observed at dataset ratio of tra:test = 4:1; 0.821 ± 0.010 in RF, 0.889 ± 0.008 in XGB, 0.893 ± 0.007 in LGBM, and 0.894 ± 0.010 in CB, respectively (Table 3). In addition, the highest mean Acc in test datasets for RF, XGB, LGB, and CB in 10-fold cross validation were 0.9043 ± 0.0032 at dataset ratio of tra:test = 2:1 in RF, 0.9121 ± 0.0037 at dataset ratio of tra:test = 4:1 in XGB, 0.9153 ± 0.0047 at dataset ratio of tra:test = 4:1 in LGBM, and0.9115 ± 0.0058 at dataset ratio of tra:test = 4:1 in in CB, respectively (Table S4). Furthermore, in order to compare the performance of prediction model by the molecular descriptors as input dataset of neural network (NN) with DeepSnap-DL, the molecular descriptors extracted from Tra and Test datasets used in DeepSnap-DL by Moerdred were applied to NN using JMP pro application. The highest mean AUC values of five kinds of dataset ratio of tra:test (1:1 to 5:1) in 10-fold cross validation were observed at dataset ratio of tra:test = 4:1; 0.842 ± 0.029 in (Table 3). Permutation test that randomly replaced activity scores of the PR in dataset ratio of tra:test = 5:1 showed non-predictive abilities: AUCs were 0.488 ± 0.038 in RF, 0.489 ± 0.032 in XGB, 0.482 ± 0.038 in LGBM, 0.473 ± 0.025 in CB, and 0.498 ± 0.035 in NN, respectively (Table 3). These results demonstrate that the DeepSnap-DL method outperformed the traditional ML methods such as RF, XGB, LGB, CB, and NN for predicting the activity of the PR antagonists. However, it remains unclear which process of the DeepSnap-DL method affects the performance. Recently, the graph, in which atoms and molecular bonds are represented by nodes and edges as feature vector has become a useful tool due to enable capturing the structural relations among input data (Jensen, 2019; Jippo et al., 2019; Takata et al., 2020). However, some problems about this have been pointed out. The complex and diverse connectivity patterns for the graph-structured data, such as non-Euclidean nature often are difficult to gain proper features (Zhang S. et al., 2019). A low-dimensional representation by embedding in a low-dimensional Euclidean space for handling such complex structures (Reutlinger and Schneider, 2012; Li B. et al., 2019). On the other hand, graph CNN (GCN) using DL, which defined convolution operations for the graph structures indicates their powerful capability by using the graph structure data directly as input data for NN due to convolutions and filtering on the non-Euclidean characteristic of graph (Coley et al., 2018; Meng and Xiang, 2018; Eguchi et al., 2019; Liu K. et al., 2019; Miyazaki et al., 2019; Ryu et al., 2019). Whereas, some drawbacks for this GCN have also been shown (Kipf and Welling, 2017; Zhou et al., 2018; Wu Z. et al., 2019). First, if the graph convolution operation was repeated by increase of the number of layers, the representation at all nodes would converge to same values, so that the performance of the GCN was decreased (Wu F. et al., 2019; Zhang S. et al., 2019). Second, most spectral-based approaches by transforming the graph into the spectral domain through the eigenvectors of the Laplacian matrix cannot be performed on graphs with different size numbers of vertices and Fourier bases (Bail et al., 2019). Third, the problem of identifying the class labels of nodes in a graph, in which if the small number of labels was used, their information cannot be propagated throughout the graph (Chen et al., 2019; Jiang et al., 2019; Wu F. et al., 2019). Fourth, since the depiction of the chemical structure by the graph simply represents a bond between atoms, the GCN lacks the interatomic distance and 3D-structual information. While, the DeepSnap-DL method can analyze the 3D-conformational sampling with multiple angles. In this study, the performance in the DeepSnap-DL using pictures as input data were higher compared with that of the NN using descriptors as input. In addition, the performances between the four conventional MLs including RF, XGB, LGBM, and CB and the NN using descriptors as input data showed no differences. These results indicate that pictures produced 3D-chemical structure may be important for building the high-performance in the DeepSnap-DL method. While, since the main factor(s) corresponding to the molecular descriptors in the constructing of the prediction model remain unknown, it is difficult to estimate their molecular actions.

**Table 2:** Prediction performances in three kinds of machine learning (ML) with 3D-chemical structures derived from nine kinds of cleaning rules using molecular descriptors extracted by MORDRED.

		**none_2D**		**none_3D**		**none_Corina**		**domi_2D**		**domi_3D**		**domi_Corina**		**neut_2D**		**neut_3D**		**neut_Corina**	
**Test dataset sizes**	**MLs**	**Means**	**SD**	**Means**	**SD**	**Means**	**SD**	**Means**	**SD**	**Means**	**SD**	**Means**	**SD**	**Means**	**SD**	**Means**	**SD**	**Means**	**SD**
0.5	RF	0.8327	0.0050	0.8275	0.0084	0.8250	0.0073	0.8275	0.0122	**0.8497**	0.0121	0.8187	0.0136	0.8214	0.0123	0.8231	0.0122	0.8135	0.0087
0.5	XGB	0.9001	0.0053	0.8926	0.0041	0.8874	0.0080	0.8868	0.0071	**0.9142**	0.0038	0.8814	0.0065	0.8880	0.0065	0.8831	0.0066	0.8834	0.0055
0.5	LGB	0.9017	0.0054	0.8951	0.0056	0.8922	0.0041	0.8924	0.0084	**0.9267**	0.0047	0.8901	0.0087	0.8954	0.0064	0.8849	0.0063	0.8902	0.0089
0.5	LGB_hyp	0.9030	0.0075	0.8960	0.0069	0.8903	0.0065	0.8906	0.0082	**0.9189**	0.0077	0.8876	0.0079	0.8922	0.0077	0.8854	0.0088	0.8901	0.0079
0.2	RF	0.8496	0.0112	0.8453	0.0161	0.8343	0.0171	0.8353	0.0169	**0.8624**	0.0140	0.8320	0.0147	0.8388	0.0223	0.8402	0.0171	0.8271	0.0110
0.2	XGB	0.9084	0.0094	0.9032	0.0141	0.8968	0.0048	0.8927	0.0114	**0.9260**	0.0061	0.8939	0.0139	0.8988	0.0090	0.8972	0.0114	0.8920	0.0123
0.2	LGB	0.9173	0.0120	0.9136	0.0116	0.9108	0.0078	0.9028	0.0076	**0.9309**	0.0093	0.9034	0.0094	0.9075	0.0086	0.9020	0.0118	0.9003	0.0111
0.2	LGB_hyp	0.9176	0.0063	0.9160	0.0083	0.9092	0.0066	0.8941	0.0142	**0.9346**	0.0069	0.8983	0.0061	0.9050	0.0092	0.8970	0.0076	0.9085	0.0075
0.1	RF	0.8557	0.0134	0.8497	0.0218	0.8220	0.0176	0.8410	0.0198	**0.8574**	0.0159	0.8539	0.0150	0.8518	0.0209	0.8462	0.0343	0.8466	0.0129
0.1	XGB	0.9153	0.0140	0.9053	0.0171	0.8944	0.0141	0.8947	0.0283	**0.9272**	0.0118	0.8970	0.0166	0.8905	0.0132	0.8976	0.0153	0.8945	0.0199
0.1	LGB	0.9185	0.0125	0.9119	0.0078	0.9019	0.0175	0.9056	0.0159	**0.9407**	0.0134	0.9031	0.0102	0.9067	0.0159	0.9150	0.0124	0.9121	0.0117
0.1	LGB_hyp	0.9206	0.0149	0.9133	0.0048	0.9069	0.0138	0.9080	0.0135	**0.9411**	0.0126	0.9060	0.0089	0.9095	0.0121	0.9155	0.0120	0.9114	0.0102

*LGB_hyp indicates the predition model by LGB optimized with HyperOpt test dataset size show that 0.5 is Tra:Test = 0.5:0.5, 0.2 is Tra:Test = 0.8:0.2, and 0.1 is Tra:Test = 0.9:0.1, respectively. Most high-performance of prediction in each cleaning rules were indicated by bold*.

**Table 3:** Prediction performances with different dataset sizes on the four Machine Learnings.

	**MLs**	**RF**		**XGB**		**LGBM**		**CB**		**NN**	
	**tra:val:test**	**Means**	**SD**	**Means**	**SD**	**Means**	**SD**	**Means**	**SD**	**Means**	**SD**
AUC	1: 1: 1	0.794	0.015	0.870	0.007	0.870	0.012	0.872	0.009	0.806	0.021
AUC	2: 2: 1	0.807	0.012	0.874	0.012	0.876	0.012	0.877	0.011	0.821	0.019
AUC	3: 3: 1	0.818	0.022	0.887	0.014	0.889	0.013	0.888	0.013	0.831	0.025
AUC	4: 4: 1	**0.821**	0.010	**0.889**	0.008	**0.893**	0.007	**0.894**	0.010	**0.842**	0.029
AUC	5: 5: 1	0.811	0.027	0.879	0.020	0.890	0.018	0.886	0.016	0.824	0.024
AUC	5: 5: 1 PMT	0.488	0.038	0.489	0.032	0.482	0.038	0.473	0.025	0.498	0.035

*Most high-peformance in each MLs were indicated bt bold*.

*MLs, RF, XGB, LGBM, CB, and NN indicate machine learnings, random forest, Xgboost, Light GBM, Cat boost, and neural network, respectively*.

In this study, we showed that the DeepSnap-DL approach enables high-throughput and high-quality prediction of the PR antagonist due to the automatic extraction of feature values from 3D-chemical structures adjusted as suitable input data into the DL, as well as avoiding overfitting through selective activation of molecular features with integration of multi-layered networks (Guo et al., 2017; Liang et al., 2017; Kong and Yu, 2018; Akbar et al., 2019). In addition, consist with recent reports (Chauhan et al., 2019; Cortés-Ciriano and Bender, 2019), this study indicated both the training data size and image redundancy are critical factors when determining prediction performance. In addition, there is long-standing problem of class imbalanced data for the MLs that resulted in low-performance by extremely different distribution of labeled input data (Haixiang et al., 2017). To resolve this issue, adjustment of sampling (over-sampling, adding repetitive data and under-sampling, removing data) and class weights in loss functions (softmax cross-entropy, sigmoid cross-entropy, and focal loss), where weights are assigned to data in order to match a given distribution, have been applied, but these countermeasures have some drawbacks such as overfitting, redundancy, valuable information loss, and how to assign weights and select loss functions (Chawla et al., 2002; Chang et al., 2013; Dubey et al., 2014; Cui et al., 2019). However, despite the excellent predictive performance, there is still room for improving the DeepSnap-DL method. First, it is still unclear what and where feature value(s) are extracted from the input image in the DeepSnap-DL process. In the DL, the features within an image are extracted by a convolution process with CNNs. Therefore, by specifying the convolutional region(s) using combination analysis with other, new methods to visualize the region(s) of the feature(s) (Selvaraju et al., 2016; Xu et al., 2017; Farahat et al., 2019; Oh et al., 2019; Xiong et al., 2019), the important part or area of the chemical structure necessary for prediction model construction could be estimated. Secondly, the optimal 3D-structuring rules have not been defined. In a recent report (Matsuzaka and Uesawa, 2019b), among ten conformational samplings of 3D-chemical structures, the combination of adjusted protonation state by the neutralized and coordinated washed species by CORINA (neut_Corina) in the MOE database construction process indicated overperformance of prediction models compared to nine conformational samplings. However, in this study, the washing treatment of the chemical structures by the neut_Corina did not represent highest prediction performance for the nine conformational samplings examined. These findings suggest that the conformational sampling method leading the best predictive performance may vary depending on the target models.

In conclusion, the DeepSnap-DL approach is a more effective ML method that could fulfill the growing demand for rapid *in silico* assessments of not only pharmaceutical chemical compounds including agonists and antagonist, but also the safety evaluations for industrial chemicals.

## Materials and Methods

### Data

The original datasets for a total of 9,667 chemical structures and the corresponding PR activity scores used in this study were downloaded as reported previously (Matsuzaka and Uesawa, 2019a,b), in the simplified molecular input line entry system (SMILES) format from the PubChem database (PubChem assay AID 1347031). The database consisted of quantitative high-throughput screening (qHTS) results for PR antagonists derived from the Tox21 10k library composed of compounds mostly procured from commercial sources, including pesticides, industrial chemicals, food additives, and drugs based on known environmental hazards, exposure concerns, physiochemical properties, commercial availability, and cost (Huang et al., 2014; Huang, 2016). Since the dataset includes some similar chemical compounds, but with different activity scores for different ID numbers due to the presence of possible stereoisomers or salts, these chemical compounds with indefinite activity criteria, non-organic compounds, and/or inaccurate SMILES were eliminated. A total of 7,582 chemicals for the PR antagonists were then chosen for a non-overlapping input dataset (Table S1). In the qHTS of the Tox21 program to identify the chemical compounds that inhibit PR signaling, the PR antagonist activity scores were determined from 0 to 100% based on a compound concentration response analysis as follows: % Activity = ((Vcompound–Vdmso)/(Vpos–Vdmso)) × 100, where Vcompound, Vdmso, and Vpos denote the compound, the median values of the DMSO only, and the median value of the positive control well values measuring by expression of a beta-lactamase reporter gene under the control of an upstream activator sequence, respectively. These were then corrected by using compound-free control plates, i.e., DMSO-only plates, at the beginning and end of the compound plate measurement (Huang et al., 2014, 2016; Huang, 2016). The Pubchem_activity_scores of the PR antagonists were grouped into the following three classes: (1) zero, (2) from 1 to 39, and (3) from 40 to 100, represented as inactive, inconclusive, and active compounds, respectively. In this study, compounds with activity scores from 40 to 100 or from 0 to 39 were defined as active (760 compounds) or inactive (6,822 compounds), respectively (Table S1). We then applied a 3D conformational import from the SMILES format using MOE 2018 software (MOLSIS Inc., Tokyo, Japan) to generate the chemical database. To determine a suitable form of each chemical structure for the building the prediction models, a database Wash application was applied. The protonation menu of the Wash application was set to neutralize and charged species were replaced if the following conditions were met: (1) all the atoms are neutral; (2) the species is neutral overall; or (3) the least charge-bearing form of the structure or dominant form is present, whereby the molecule was replaced with the dominant promoter/tautomer at pH 7 used in this study. In addition, the coordinates of the washed species were adjusted based on the following conditions: (1) the results of the 2D depiction layout algorithm if Depict 2D was selected; (2) those generated by a cyclic 3D embedder based on distance geometry and refinement if Rebuild 3D is selected; or (3) those generated by the external program, CORINA classic software (Molecular Networks GmbH, Nürnberg, Germany, https://www.mn-am.com/products/corina). The nine types of combinations of the protonation states (none, dominant, neutralize) and coordinating washed species (depict 2D, rebuild 3D, CORINA) when washing the MOE database were investigated: none_2D (none, depict 2D), domi_2D (dominant, depict 2D), neut_2D (neutralize, depict 2D), none_3D (none, rebuild 3D), domi_3D (dominant, rebuild 3D), neut_3D (neutralize, rebuild 3D), none_CORINA (none, CORINA), domi_CORINA (dominant, CORINA), and neut_CORINA (neutralize, CORINA). The 3D structures were finally saved in the SDF file format as described previously (Agrafiotis et al., 2007; Chen and Foloppe, 2008; Matsuzaka and Uesawa, 2019a,b) To scrutinize how to divide the dataset, we performed a permutation test for the activity scores randomly labeled as all chemical compounds. The dataset was split into N groups, where N is Rt + Rv + 1 (Rt and Rv were integers for ratio of Tra and Val datasets). Three dataset groups, including Tra, Val, and Test, were then built with a Rt: Rv: 1 ratio from N groups of datasets. A prediction model was created by Tra ad Val datasets, and scrutinized the performance with Test dataset. Finally, we calculated prediction performance using the Test dataset. In the following analysis, the other test dataset was selected from a group that was not used in the first analysis. The model was built and its calculation of probability was examined in the same manner. When the N-times analysis was completed, a new N-segment dataset was prepared. Similarly, the model was constructed and its performance was evaluated. Finally, a total of ten tests were performed that is *N*-fold cross validation, in which this study used *N* = 10 for reducing the bias (Moss et al., 2018).

### DeepSnap

Using the SDF files prepared by the MOE application, the 3D chemical structures of the PR antagonist compounds were depicted as 3D ball-and-stick models by a Jmol, an open-source Java viewer software for 3D molecular modeling of chemical structures (Hanson, 2016; Scalfani et al., 2016; Hanson and Lu, 2017). The 3D-chemical models were captured automatically as snapshots of user-defined angle increments on the x-, y-, and z-axes, which were saved as 256 × 256 pixel resolution PNG files (RGB) and split into three types of datasets, Tra, Val, and Test datasets, as previously reported (Matsuzaka and Uesawa, 2019a,b). To design suitable molecular images for their classifications at the next step, some parameters during the DeepSnap depiction process, such as image pixel size, image format (png or jpg), molecule number per SDF file to split into (MPS), zoom factor (ZF, %), atom size for van der Waals radius (AT, %), bond radius (BR, mÅ), minimum bond distance (MBD), and bond tolerance (BT) were set based on the previous study (Matsuzaka and Uesawa, 2019a,b), and background colors (BC) was examined in this study. Of these parameters, six BCs including black (0, 0, 0), white (255, 255, 255), red (255, 0, 0), yellow (255, 255, 0), green (0, 255, 0), and blue (0, 0, 255) were examined. To investigate the combinations of pictures with prediction performances, two, three, and four pictures were randomly selected from eight pictures produced at angle 300°, after which 10 combinations of pictures were prepared for the following allocations: two pictures: [(0,0,0), (0,300,0)], [(0,0,0), (0,0,300)], [(0,0,0), (300,0,0)], [(0,0,0), (300,300,0)], [(0,0,0), (300,300,300)], [(0,0,300), (0,300,0)], [(0,0,300), (300,300,0)], [(0,0,300), (0,300,300)], [(0,300,0), (0,300,300)], [(0,300,0), (300,300,300)]; three pictures, [(0,0,0), (0,300,0), (0,0,300)], [(0,0,0), (0,300,0), (0,300,300)], [(0,0,0), (0,300,300), (300,300,300)], [(0,0,0), (300,0,300), (300,300,300)], [(0,0,300), (300,0,0), (300,300,300)], [(0,0,300), (300,0,300), (300,300,300)], [(0,0,300), (0,300,0), (300,300,300)], [(0,300,0), (300,300,0), (300,300,300)], [(0,300,300), (0,300,300), (300,300,0)], [(0,300,300), (300,0,0), (300,300,300)]; and four pictures, [(0,0,0), (0,0,300), (0,300,0), (300,0,0)], [(0,300,300), (300,0,300), (300,300,0), (300,300,300)], [(0,0,0), (0,300,300), (300,0,0), (300,300,300)], [(0,0,300), (300,0,300), (300,300,0), (300,0,0)], [(0,0,0), (300,0,0), (300,300,0), (300,300,300)], [(0,0,300), (0,300,0), (300,0,0), (300,0,300)], [(0,0,300), (0,300,0), (300,300,300), (0,0,300)], [(0,300,0), (300,300,0), (300,300,300), (0,0,300)], [(0,300,300), (0,300,300), (300,300,0), (300,0,300)], [(0,300,300), (300,0,0), (300,300,300), (300,300,300)].

### ML Models

The following four different ML models were chosen to construct the prediction models for PR antagonist activity: (1) DL, (2) RF, (3) XGB, and (4) LightGBM. For the (1) DL, all the PNG image files produced by DeepSnap were resized by utilizing NVIDIA DL GPU Training System (DIGITS) version 4.0.0 software (NVIDIA, Santa Clara, CA, USA), on four-GPU systems, Tesla-V100-PCIE (31.7 GB) with a resolution of 256 × 256 pixels as input data, as previously reported (Matsuzaka and Uesawa, 2019a,b). To rapidly train and fine-tune the highly accurate DNNs using the input Tra and Val datasets based on the image classification and building the prediction model pre-trained by using ILSVRC (ImageNet Large Scale Visual Recognition Challenge) 2012 dataset (http://image-net.org/challenges/LSVRC/2012/browse-synsets) including 1,000 class names such as animal (40%), device (12%), container (9%), consumer goods (6%), equipment (4%), etc., that split into 1.2 million of train, fifty thousand of Val, one million of Test datasets extracted from ImageNet (http://www.image-net.org/index), as transfer learning (Matsuzaka and Uesawa, 2019a,b), we used a pre-trained open-source DL model, Caffe, and the open-source software on the CentOS Linux distribution 7.3.1611. In this study, the network of GoogLeNet was used deep CNN architectures comprised complex inspired by LeNet, and implemented a novel module called “Inception,” which used batch normalization, image distortions, and RMSprop, and concatenates different filter sizes and dimensions into a single new filter and introduces sparsity and multiscale information in one block (Figure S9). There is a 22 layer deep CNN, comprised of two convolutional layers, two kinds of pooling layers (four max pools and one avg pool), and nine “Inception” modules, in which each module has six convolution layers and one pooling layer, and 4 million of parameters (Table S5; Szegedy et al., 2014; Yang et al., 2018; Kim J. Y. et al., 2019). At the DeepSnap-DL method, the prediction models were constructed by training datasets using 30 of epochs in DL. Among these epochs, most low of Loss value in Val dataset was selected for next examination to prediction using Test dataset.

For the (2) RF based on decision trees, where each tree is independently constructed and each node is split using the best among the subset of predictors randomly chosen at the node, (3) XGB combined weak learners (decision trees) to achieve stronger overall class discrimination, and (4) LightGBM modified gradient boosting algorithm by gradient-based one-side sampling and exclusive feature bundling, molecular descriptors were calculated using a Python package Mordred (https://github.com/mordred-descriptor/mordred) (Moriwaki et al., 2018). Classification experiments were conducted in the Python programming language using specific classifier implementations, RF (https://github.com/topics/random-forest-classifier), XGB (https://github.com/dmlc/xgboost/tree/master/python-package), and LightGBM (https://github.com/microsoft/LightGBM) provided by the scikit-learn and rdkit Python packages (Czodrowski, 2013; Chen and Guestrin, 2016; Ke et al., 2017; Kotsampasakou et al., 2017; Sandino et al., 2018; Zhang J. et al., 2019), as previously reported (Matsuzaka and Uesawa, 2019a,b). In addition, the prediction models build by LightGBM were optimized by Hyperopt, which is a python library for the sequential model-based optimization (also Bayesian optimization) of hyperparameters of ML algorithms (https://github.com/hyperopt/hyperopt). As for dataset split, all chemical compounds were randomly separated into two Tra and Test datasets using train_test_split function (test_size = 0.5, 0.2, 0.1).

### Evaluation of the Predictive Model

Using 10-fold cross validation in the DL prediction model, we analyzed the probability of the prediction results using the prediction model with the lowest minimum Loss in Val value among 30 examined echoes. Since we calculated the probabilities for each image prepared from different angles with the *x*-, *y*-, and *z*-axes directions calculated for one molecule during the process of the DeepSnap-DL method, the medians of each these predicted values were used as the representative values for target molecules, as described previously (Matsuzaka and Uesawa, 2019a,b). Classification performance was evaluation using information retrieved from confusion matrix. Based on the sensitivity (Equation 1), which is a true positive rate identified as positive for all the positive samples including true and false positives, and the specificity (Equation 2), which is a true negative rate identified as negative for all the negative samples including true and false negatives, a confusion matrix regarding the predicted and experimentally defined labels was used to make the ROC curve and calculate the AUC using JMP Pro 14, which is a statistical discovery software (SAS Institute Inc., Cary, NC, USA), as reported previously (Matsuzaka and Uesawa, 2019a,b). Therefore, it follows that where TP, FN, TN, and FP denote true positive, false negative, true negative, and false positive, respectively:

(1) $$\begin{array}{l} \text{Sensitivity}=\sum \text{TPs}/\left(\sum \text{TPs}+\sum \text{FNs}\right) \end{array}$$

(2) $$\begin{array}{l} \text{Specificity}=\sum \text{TNs}/\left(\sum \text{TNs}+\sum \text{FPs}\right) \end{array}$$

Additionally, since the proportion between the “active” and “inactive” compounds for the activity scores is biased in the Tox21 10k library (Huang et al., 2016), the BAC (Equation 3), Acc (Equation 4), Precision (Equation 5), Recall (Equation 6), *F* value (Equation 7), and MCC (Equation 8) were utilized to properly evaluate imbalanced data by applying a cut-off point calculated using the JMP Pro 14 and statistical discovery software.

(3) $$\begin{array}{l} \text{BAC}=\left(\text{sensitivity}+\text{specificity}\right)/2 \end{array}$$

(4) $$\begin{array}{l} \text{Accuracy}=\left(\text{TP + TN}\right)/\left(\text{TP + FP + TN + FN}\right) \end{array}$$

(5) $$\begin{array}{l} \text{Precision}=\text{TP}/\left(\text{TP}+\text{FP}\right) \end{array}$$

(6) $$\begin{array}{l} \text{Recall}=\text{TP}/\left(\text{TP}+\text{FN}\right) \end{array}$$

(7) $$\begin{array}{l} \text{F}\text{value}=2\times \text{Recall}\times \text{Precision/}\left(\text{Recall }+\text{ Precision}\right) \end{array}$$

(8) $$\begin{array}{lll} \text{MCC}=\left(\text{TP}\times \text{TN}-\text{FP}\times \text{FN}\right)\\ \; & \; & /\sqrt{\left\{\left(\text{TP}+\text{FP}\right)\times \left(\text{TP}+\text{FN}\right)\times \left(\text{TN}+\text{FP}\right)\times \left(\text{TN + FN}\right)\right\}} \end{array}$$

For RF, XGB, and LGB, we calculated the AUC using Python 3 and open source ML libraries, including scikit-learn (Pedregosa et al., 2011; Kensert et al., 2018). Differences in mean levels of performance for combinations of different angles in DeepSnap. Difference between mean levels of performance, including AUC, BAC, F, MCC, Loss.Val, Acc.Test, and Acc.Val, for one combination and rest nine combinations for pick-up pictures from eight pictures produced at angle 300° were indicated as Delta_AUC, Delta_BAC, Delta_F, Delta_MCC, Delta_ Loss.Val, Delta_ Acc.Test, and Delta_ Acc.Val, respectively with 95% CI calculated by Microsoft Excel 2016.

### PCA

PCA of the molecular descriptors extracted from a total 7,581 of chemical compounds was performed by using JMP Pro 14. Each set of 687 molecular descriptors derived from a total of nine SDF files produced based on the cleaning rules, including protonation and coordinates, were analyzed to represent multivariate information in a reduced subspace of principal components (PCs). Eigenvalues, which represent the amount of variation explained by each PCs were calculated and the rates of the variation explained by each PCs, whose scores obtained by linear combination of variables with eigenvector weights, were displayed as bar graph according to user's guide (SAS Institute Inc., 2018).

### Statistical Analysis

Differences in prediction performances, including loss (Val), Acc (Val), BAC, F, AUC, Acc (Test), and MCC were analyzed by the Mann–Whitney U test (Chakraborty and Chaudhuri, 2014; Dehling et al., 2015; Dedecker and Saulière, 2017). Finding of corrected P (Pc) < 0.05 is significant based on corrections from multiple testing, such as the Bonferroni's method.

## Data Availability Statement

All datasets generated for this study are included in the article/Supplementary Material.

## Author Contributions

YU initiated and supervised the work, designed the experiments, collected the information about chemical compounds, and edited the manuscript. YM performed the computer analysis, the statistical analysis, and drafted the manuscript. YU and YM read and approved the final manuscript.

### Conflict of Interest

The authors declare that the research was conducted in the absence of any commercial or financial relationships that could be construed as a potential conflict of interest.

## Supplementary Material

Figure S1 Prediction performances with combinations of different angles in the DeepSnap. Two (blue boxes in right), three (yellow boxes in middle), and four (green boxes in left) of pictures were randomly selected from eight pictures produced at angle 300°, and after which ten picture combinations were prepared. The means BAC **(A)**, *F* **(B)**, Acc(Val) **(C)**, and Loss(Val) **(D)** were calculated by 10-fold cross validation. ^*^Pc < 0.05, ^**^Pc < 0.01, ^***^Pc < 0.001.

Figure S2 Differences in mean levels of performance for combinations of different angles in DeepSnap. Difference between mean levels of performance of one combination and rest nine combinations for pick-up pictures from eight pictures produced at angle 300° in Figure 1 were shown as blue dots with 95% confident interval (95% CI) as error bars. **(A)** Delta_BAC (95% CI), **(B)** Delta F (95% CI), **(C)** Delta_Acc.Val (95% CI), and **(D)** Delta_Loss.Val (95% CI) were calculated based on results in Figures S1A–D.

Figure S3 Performance contribution of prediction models with learning rates. The means of **(A)** Loss(Val), **(B)** Acc(Val), **(C)** BAC, and **(D)** F were calculated by 10-fold cross validation in the DeepSnap-DL-build prediction models using images produced by DeepSnap with two angles, 300 and 360°, with a Tra:Val:Test ratio of 5:5:1.

Figure S4 Performance contribution of prediction models with batch sizes (BSs). The means of **(A)** Loss(Val), **(B)** Acc(Val), **(C)** BAC, and **(D)** *F* were calculated for ten BSs from 5 to 320 by 10-fold cross validation in the DeepSnap-DL-build prediction models using images produced by DeepSnap for two angles, 300 and 360°, with a Tra:Val:Test ratio of 5:5:1.

Figure S5 Performance contribution of prediction models with solver types (STs). The means of **(A)** Loss(Val), **(B)** Acc(Val), **(C)** BAC, and **(D)** *F* were calculated for six STs (AdaDelta, AdaGrad, Adam, NAG, RMSprop, and SGD) by 10-fold cross validation in the DeepSnap-DL-build prediction models using images produced by DeepSnap for angle 300° with a Tra:Val:Test ratio of 5:5:1. ^*^Pc < 0.05, ^***^Pc < 0.001.

Figure S6 Performance contribution of prediction models with background image colors. The means of **(A)** Loss(Val), **(B)** Acc(Val), **(C)** BAC, and **(D)** *F* were calculated for six background colors (white, red, yellow, green, blue, and black) of images produced by DeepSnap for angles 300 and 360° by 10-fold cross validation in the DeepSnap-DL-build prediction models with a Tra:Val:Test ratio of 5:5:1. ^*^Pc < 0.05, ^**^Pc < 0.01, ^***^Pc < 0.001.

Figure S7 Performance contribution of prediction models with different wash conditions for preparation of chemical structures using molecular operating environment (MOE) software. For the preparation of 3D chemical structures by MOE software, combinations of three kinds of protonation (none, dominate, neutralize) and three kinds of coordinate (2D, 3D, CORINA) were used. The means of **(A)** BAC, **(B)** F, **(C)** Loss(Val), and **(D)** Acc(Val) were calculated for nine combinations of wash conditions (none_2D, none_3D, none_Corina, domi_2D, domi_3D, domi_Corina, neut_2D, neut_3D, and neut_Corina) for images produced by DeepSnap for two angles, 300 and 360°, by 10-fold cross validation with a Tra:Val:Test ratio of 5:5:1. ^*^Pc < 0.05, ^**^Pc < 0.01, ^***^Pc < 0.001.

Figure S8 Principal component (PC) analysis of 687 molecular descriptors extracted by MORDRED in nine combinations of wash conditions (none_2D, none_3D, none_Corina, domi_2D, domi_3D, domi_Corina, neut_2D, neut_3D, and neut_Corina). **(A)** Individual plots for all descriptors. **(B)** Correlation between descriptors and first principal plane (PC1 + PC2). **(C)** Eigenvalues, contribution rate (CR), and cumulative CR od PC1 to PC10.

Figure S9 The architecture of the CNN model in GoogLeNet. The pre-trained CNN comprises a 22- layer DNN: **(A)** implemented with a novel element that is dubbed an inception module; and **(B)** implemented with batch normalization, image distortions, and RMSprop, including a total of 4 million parameters.
