# Supplementary figures and images for: DeepSnap-Deep Learning Approach Predicts Progesterone Receptor Antagonist Activity With High Performance
Source: Front Bioeng Biotechnol. 2020 Jan 22;7:485. doi: 10.3389/fbioe.2019.00485 (PMC6987043; doi:10.3389/fbioe.2019.00485)

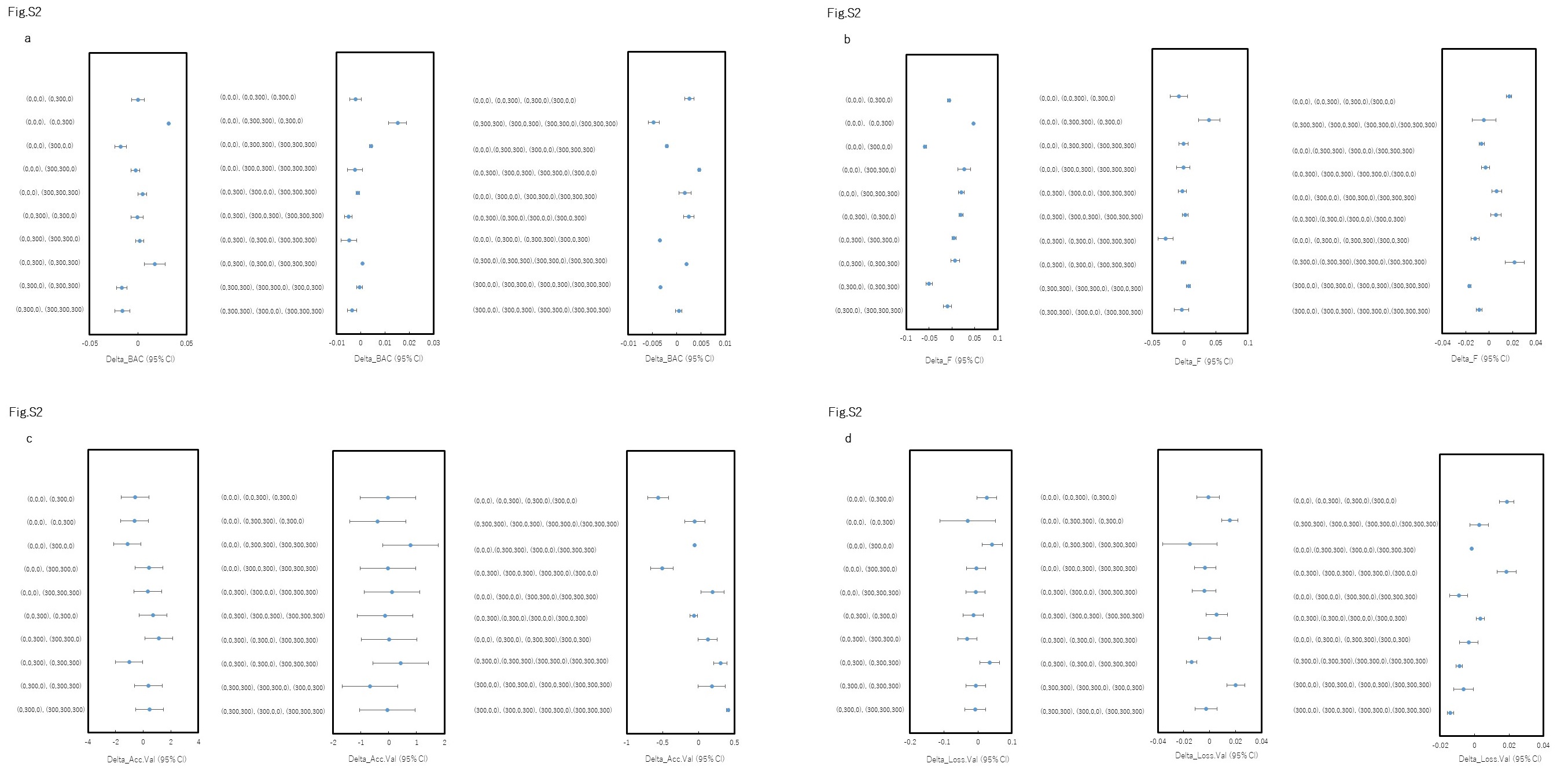

Supplement: Figure S1 — Prediction performances with combinations of different angles in the DeepSnap. Two (blue boxes in right), three (yellow boxes in middle), and four (green boxes in left) of pictures were randomly selected from eight pictures produced at angle 300°, and after which ten picture combinations were prepared. The means BAC (A), F (B), Acc(Val) (C), and Loss(Val) (D) were calculated by 10-fold cross validation. *Pc < 0.05, **Pc < 0.01, ***Pc < 0.001. [file Image_1.jpeg]

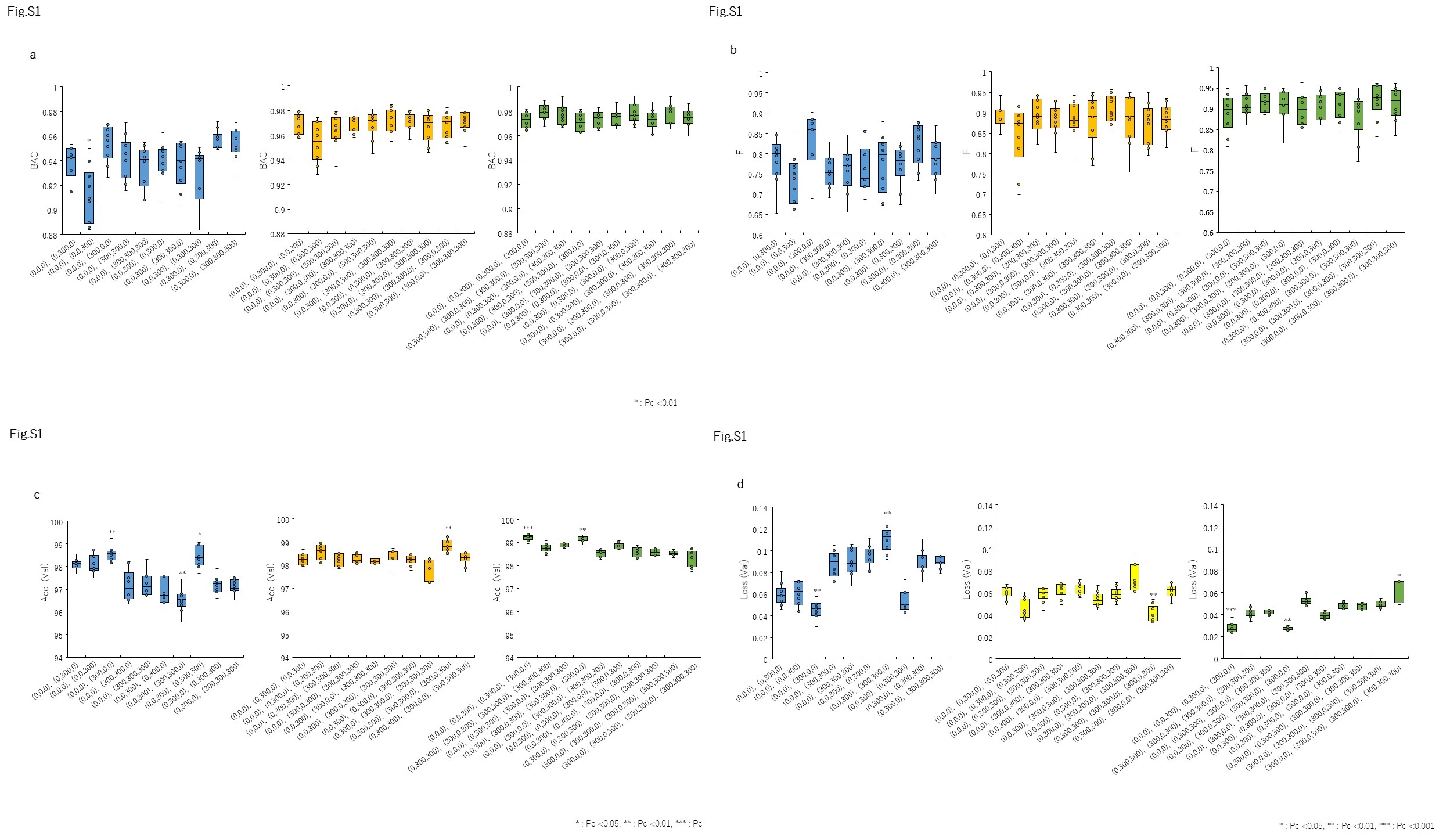

Supplement: Figure S2 — Differences in mean levels of performance for combinations of different angles in DeepSnap. Difference between mean levels of performance of one combination and rest nine combinations for pick-up pictures from eight pictures produced at angle 300° in Figure 1 were shown as blue dots with 95% confident interval (95% CI) as error bars. (A) Delta_BAC (95% CI), (B) Delta F (95% CI), (C) Delta_Acc.Val (95% CI), and (D) Delta_Loss.Val (95% CI) were calculated based on results in Figures S1A–D. [file Image_2.jpeg]

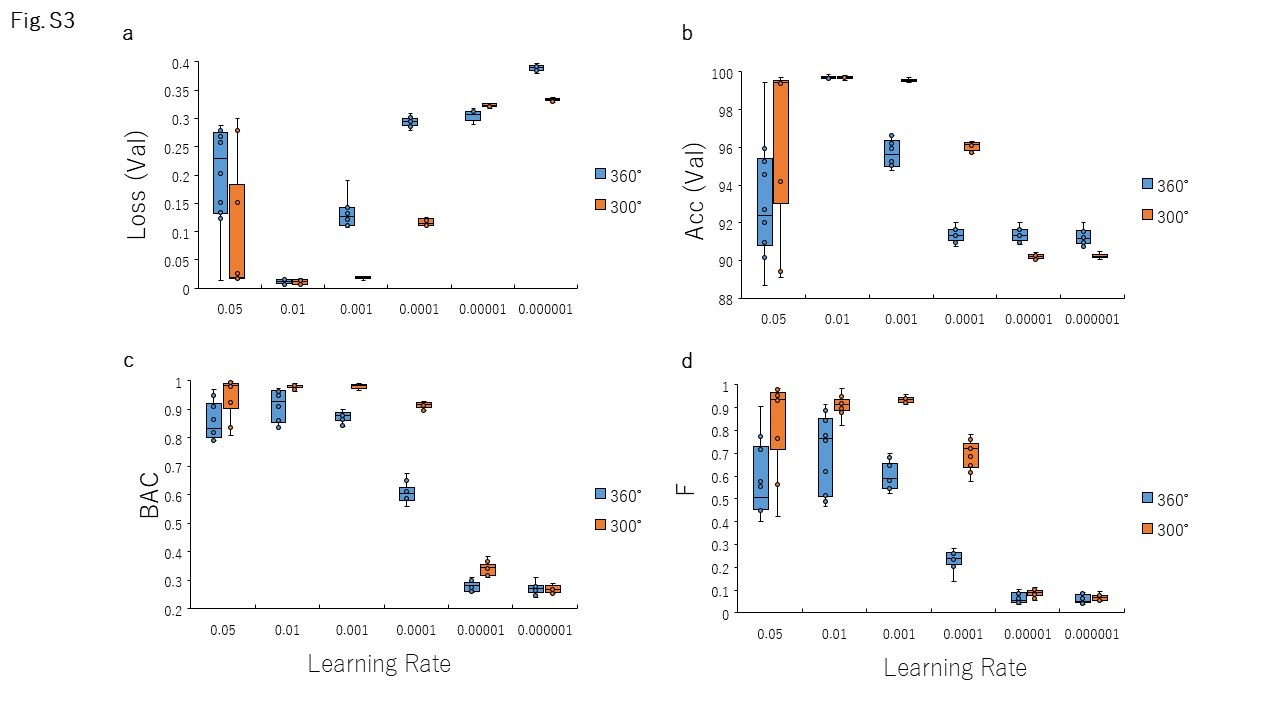

Supplement: Figure S3 — Performance contribution of prediction models with learning rates. The means of (A) Loss(Val), (B) Acc(Val), (C) BAC, and (D) F were calculated by 10-fold cross validation in the DeepSnap-DL-build prediction models using images produced by DeepSnap with two angles, 300 and 360°, with a Tra:Val:Test ratio of 5:5:1. [file Image_3.jpeg]

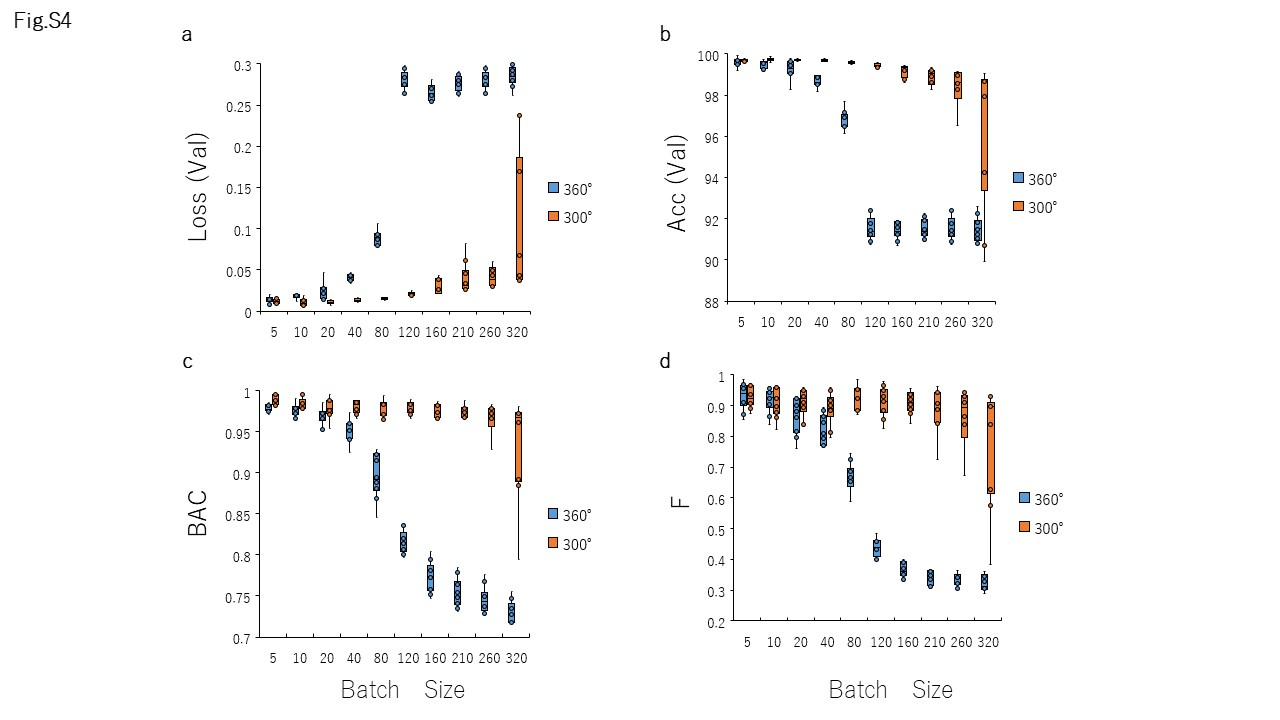

Supplement: Figure S4 — Performance contribution of prediction models with batch sizes (BSs). The means of (A) Loss(Val), (B) Acc(Val), (C) BAC, and (D) F were calculated for ten BSs from 5 to 320 by 10-fold cross validation in the DeepSnap-DL-build prediction models using images produced by DeepSnap for two angles, 300 and 360°, with a Tra:Val:Test ratio of 5:5:1. [file Image_4.jpeg]

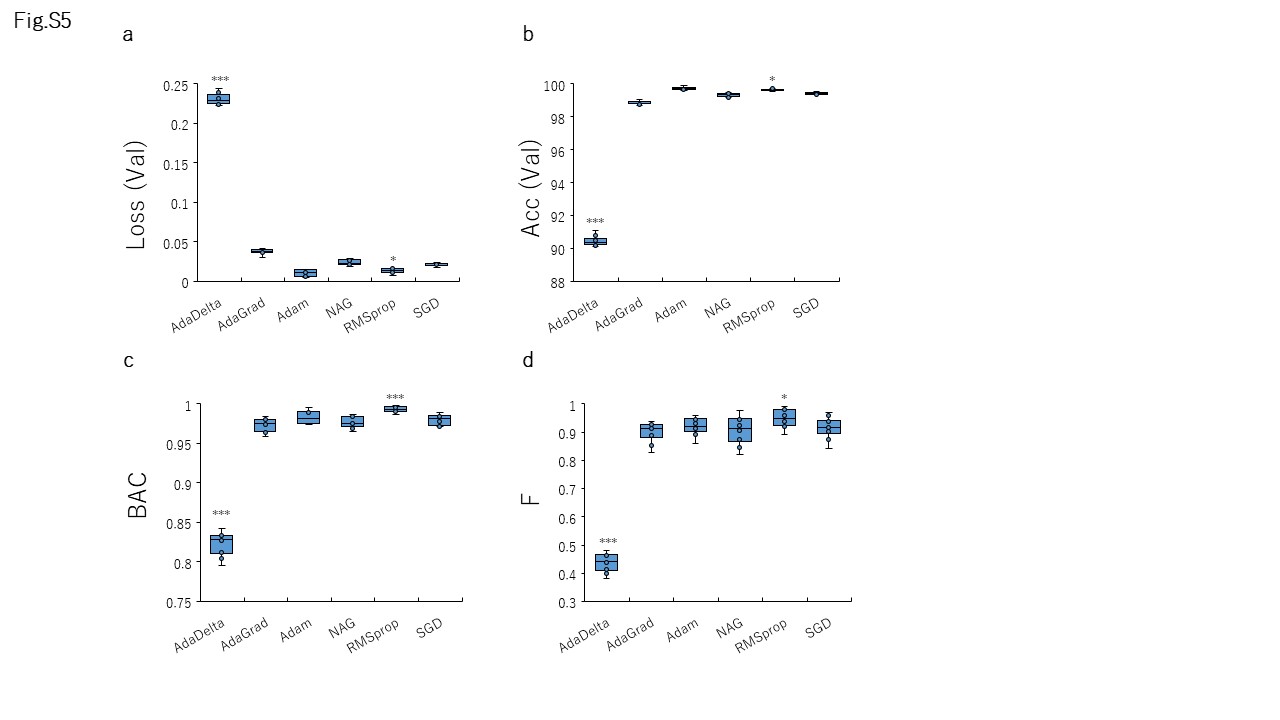

Supplement: Figure S5 — Performance contribution of prediction models with solver types (STs). The means of (A) Loss(Val), (B) Acc(Val), (C) BAC, and (D) F were calculated for six STs (AdaDelta, AdaGrad, Adam, NAG, RMSprop, and SGD) by 10-fold cross validation in the DeepSnap-DL-build prediction models using images produced by DeepSnap for angle 300° with a Tra:Val:Test ratio of 5:5:1. *Pc < 0.05, ***Pc < 0.001. [file Image_5.jpeg]

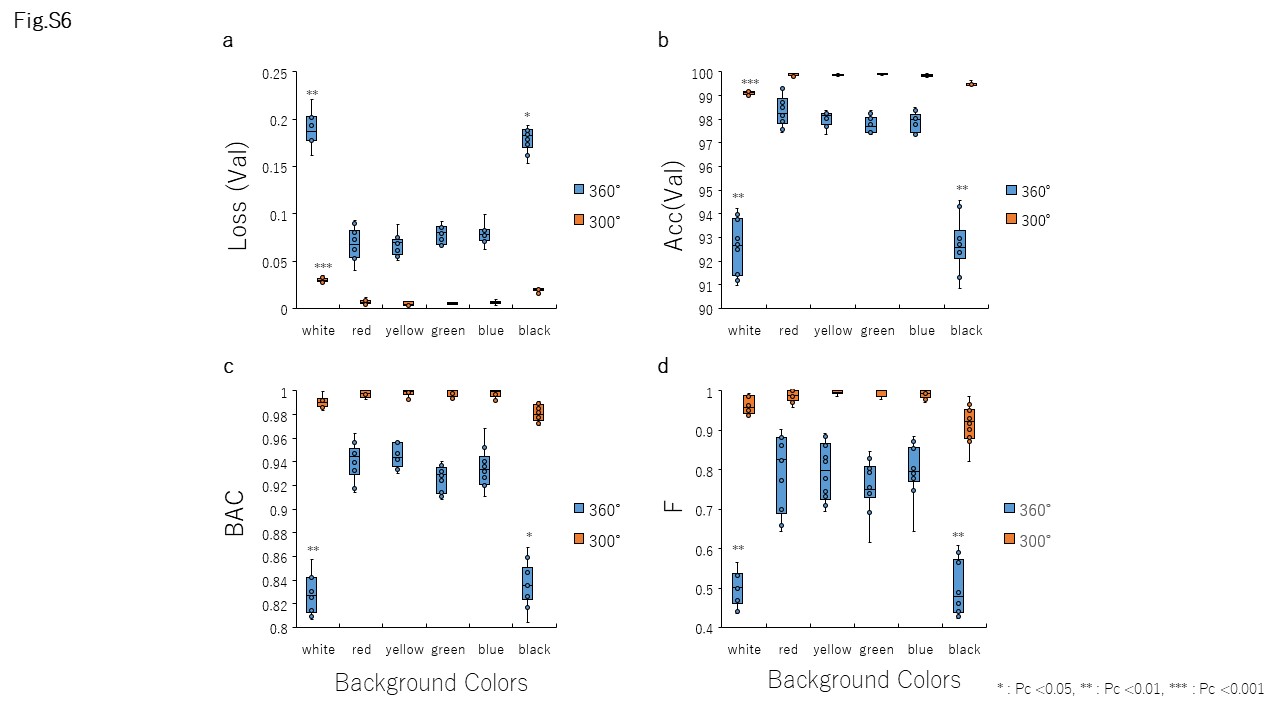

Supplement: Figure S6 — Performance contribution of prediction models with background image colors. The means of (A) Loss(Val), (B) Acc(Val), (C) BAC, and (D) F were calculated for six background colors (white, red, yellow, green, blue, and black) of images produced by DeepSnap for angles 300 and 360° by 10-fold cross validation in the DeepSnap-DL-build prediction models with a Tra:Val:Test ratio of 5:5:1. *Pc < 0.05, **Pc < 0.01, ***Pc < 0.001. [file Image_6.jpeg]

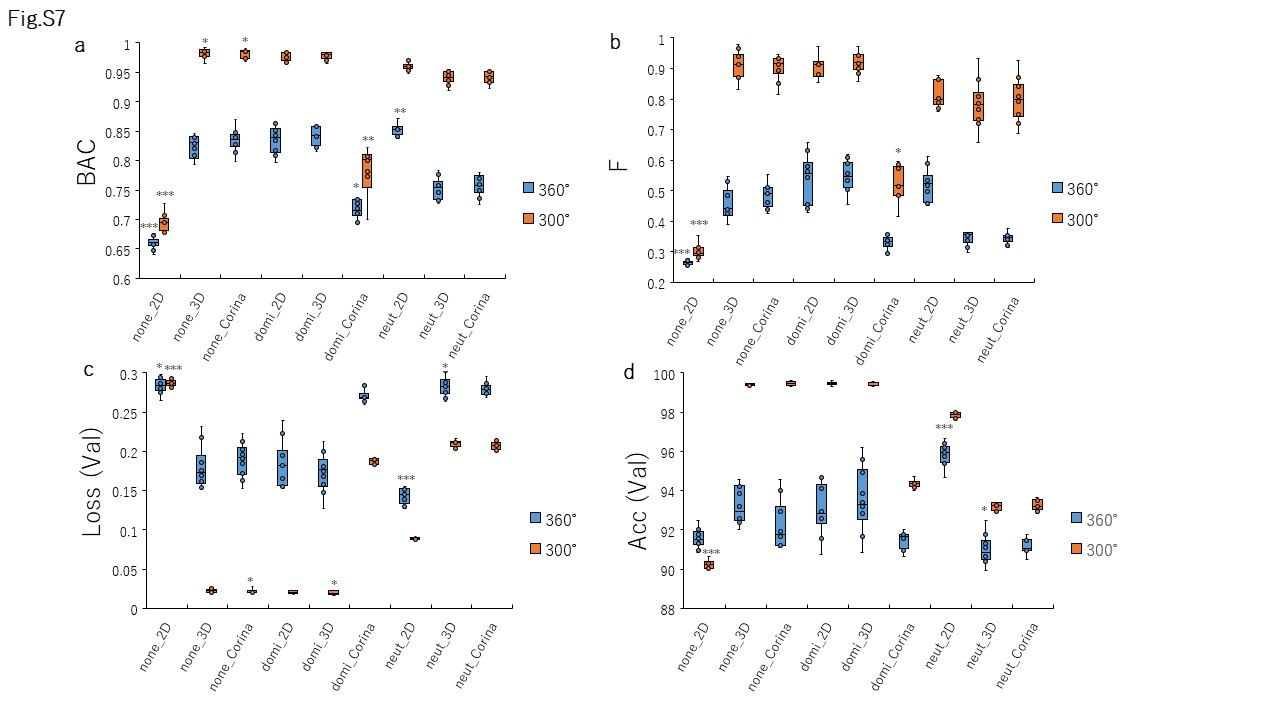

Supplement: Figure S7 — Performance contribution of prediction models with different wash conditions for preparation of chemical structures using molecular operating environment (MOE) software. For the preparation of 3D chemical structures by MOE software, combinations of three kinds of protonation (none, dominate, neutralize) and three kinds of coordinate (2D, 3D, CORINA) were used. The means of (A) BAC, (B) F, (C) Loss(Val), and (D) Acc(Val) were calculated for nine combinations of wash conditions (none_2D, none_3D, none_Corina, domi_2D, domi_3D, domi_Corina, neut_2D, neut_3D, and neut_Corina) for images produced by DeepSnap for two angles, 300 and 360°, by 10-fold cross validation with a Tra:Val:Test ratio of 5:5:1. *Pc < 0.05, **Pc < 0.01, ***Pc < 0.001. [file Image_7.jpeg]

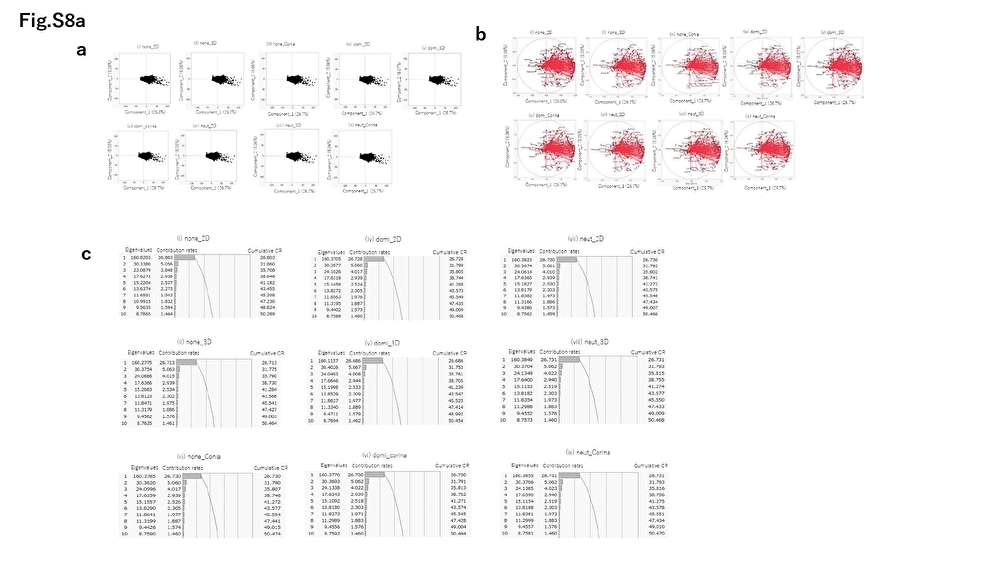

Supplement: Figure S8 — Principal component (PC) analysis of 687 molecular descriptors extracted by MORDRED in nine combinations of wash conditions (none_2D, none_3D, none_Corina, domi_2D, domi_3D, domi_Corina, neut_2D, neut_3D, and neut_Corina). (A) Individual plots for all descriptors. (B) Correlation between descriptors and first principal plane (PC1 + PC2). (C) Eigenvalues, contribution rate (CR), and cumulative CR od PC1 to PC10. [file Image_8.jpg]

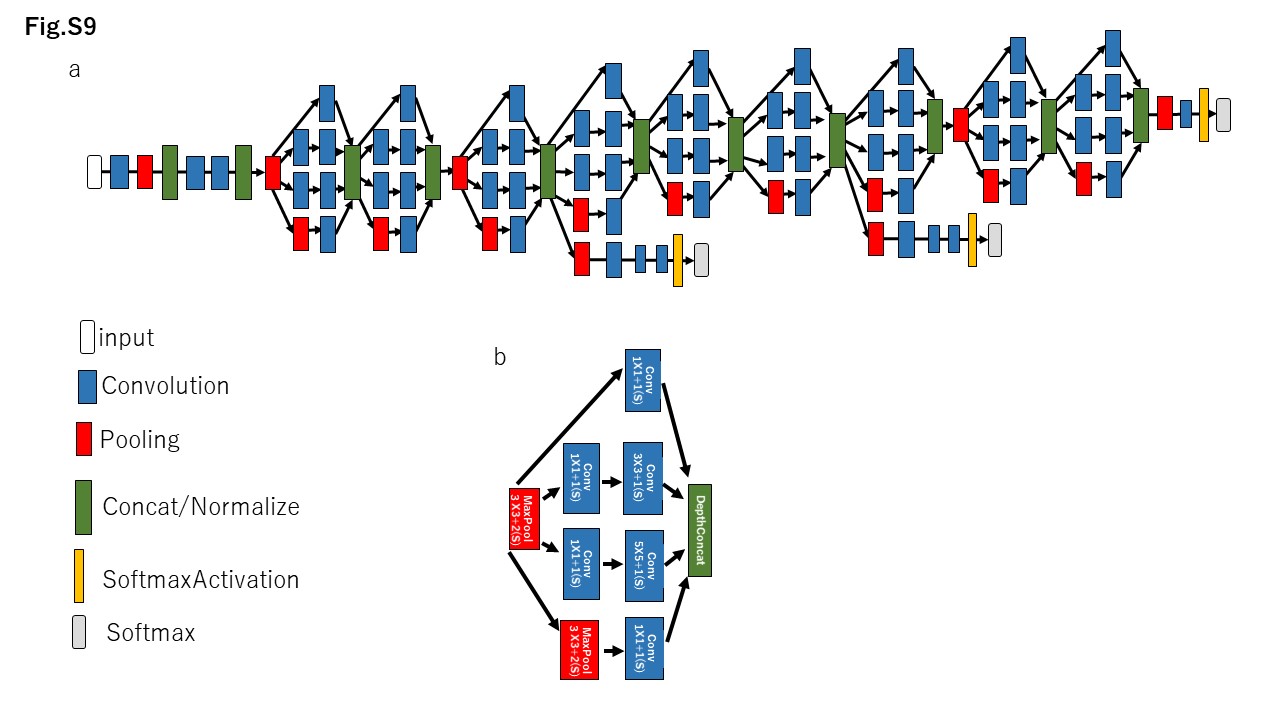

Supplement: Figure S9 — The architecture of the CNN model in GoogLeNet. The pre-trained CNN comprises a 22- layer DNN: (A) implemented with a novel element that is dubbed an inception module; and (B) implemented with batch normalization, image distortions, and RMSprop, including a total of 4 million parameters. [file Image_9.jpeg]
